# The landscape of inherited and *de novo *copy number variants in a *plasmodium falciparum *genetic cross

**DOI:** 10.1186/1471-2164-12-457

**Published:** 2011-09-22

**Authors:** Upeka Samarakoon, Joseph M Gonzales, Jigar J Patel, Asako Tan, Lisa Checkley, Michael T Ferdig

**Affiliations:** 1 Department of Biological Sciences, Eck Institute for Global Health, University of Notre Dame, Notre Dame, IN 46556, USA; 2 Roche NimbleGen, Inc., 500 S Rosa Rd, Madison, WI 53719, USA; 3 Illumina, Inc., 9885 Towne Centre Drive, San Diego, CA 92121, USA

## Abstract

**Background:**

Copy number is a major source of genome variation with important evolutionary implications. Consequently, it is essential to determine copy number variant (CNV) behavior, distributions and frequencies across genomes to understand their origins in both evolutionary and generational time frames. We use comparative genomic hybridization (CGH) microarray and the resolution provided by a segregating population of cloned progeny lines of the malaria parasite, *Plasmodium falciparum*, to identify and analyze the inheritance of 170 genome-wide CNVs.

**Results:**

We describe CNVs in progeny clones derived from both Mendelian (i.e. inherited) and non-Mendelian mechanisms. Forty-five CNVs were present in the parent lines and segregated in the progeny population. Furthermore, extensive variation that did not conform to strict Mendelian inheritance patterns was observed. 124 CNVs were called in one or more progeny but in neither parent: we observed CNVs in more than one progeny clone that were not identified in either parent, located more frequently in the telomeric-subtelomeric regions of chromosomes and singleton *de novo *CNVs distributed evenly throughout the genome. Linkage analysis of CNVs revealed dynamic copy number fluctuations and suggested mechanisms that could have generated them. Five of 12 previously identified expression quantitative trait loci (eQTL) hotspots coincide with CNVs, demonstrating the potential for broad influence of CNV on the transcriptional program and phenotypic variation.

**Conclusions:**

CNVs are a significant source of segregating and *de novo *genome variation involving hundreds of genes. Examination of progeny genome segments provides a framework to assess the extent and possible origins of CNVs. This segregating genetic system reveals the breadth, distribution and dynamics of CNVs in a surprisingly plastic parasite genome, providing a new perspective on the sources of diversity in parasite populations.

## Background

The once dominant focus on single nucleotide polymorphisms (SNPs) has given way to the recognition of a wide variety of abundant structural variants, including large and small copy number variations (CNVs) in DNA from human and chimpanzee [1-3], a range of vertebrate [4-14] and invertebrate species such as *Candida albicans *[15], *Saccharomyces cerevisiae *[16,17], as well as the malaria parasite, *Plasmodium falciparum *[18-24]. CNVs range from relatively small (≤ 1 kb or less) to more than a megabase, and include deletions, insertions, duplications/amplifications, gene conversions, and products of non-allelic homologous recombination (NAHR); affecting more total base pairs than SNPs [25]. Studies in humans and other mammals demonstrate the critical role of CNVs in generating phenotypic diversity, and disease [26,27] emphasizing the need to assess, catalogue, and understand the full spectrum of these variants. Recent studies comparing CNVs between various primate species support a contribution of CNVs to human evolution [3,28,29]; however, the role of CNVs as a source for selection has traditionally been overshadowed by the assumption that CNVs carry a high fitness cost due to altered gene dosages [30-32]. In addition to altered gene dosage, CNVs can impact genome function by disrupting coding sequences and by exerting long range (*trans*) influence on gene expression [33].

Although the earliest evidence for the impact of a CNV linked to phenotypic variation was discovered seventy years ago in *Drosophila melanogaster *[34], CNVs have been understudied largely due to the difficulties in identifying large structural polymorphisms and the presumed significance of SNPs in generating phenotypic diversity. The advent of comparative genomic hybridization (CGH) [35] and the expansion of this technique with new microarray platforms [36,37] provide rapid discovery and high-resolution, genome-wide views of CNVs.

It is well known that an abundance of structural polymorphisms in malaria parasites contribute to phenotypic diversity. Chromosome size polymorphisms have been identified in various geographical isolates, *in vitro *drug selections and controlled genetic crosses by pulse field gel electrophoresis (PFGE) [38-41]. Duplications and inter-chromosomal transpositions of chromosome segments are thought to contribute to novel phenotypes [42-46]; chromosomal anomalies, e.g. the amplification of the *pfmdr1 *(PFE1150w) locus on chromosome (Chr) 5 [47], and the deletion of the KAHRP (PFB0100c) locus on Chr 2 [48] have been studied widely for their key roles in drug resistance and cytoadherence, respectively. More recently, CNVs in *P. falciparum *have been studied in field isolates and laboratory adapted lines using various CGH platforms [18-20,22-24]. These initially relied on expression microarray designs targeting open reading frames (ORFs), while more recent experiments use densely tiled probe sets across the genome [23].

Despite the growing catalog of CNVs for various organisms, relatively little is known about their origins, stability, and inheritance. The rate at which new variants arise and/or revert to their original state, and their distribution in the genome remain largely unknown [49]. CNVs arising *de novo *are postulated to occur frequently in mammalian genomes [49-53], sometimes at higher rates than point mutations [54] and account for a more significant amount of human genetic variation [55]. A deeper understanding of CNVs, including their origins and maintenance as well as their phenotypic effects, will improve our understanding of their adaptive relevance to parasite phenotypes such as drug resistance and virulence.

Haploid progeny parasite clones derived from a genetic cross between two parent clones (HB3 × Dd2) with distinct drug-selection histories was central to mapping the molecular determinant of chloroquine (CQ) resistance [56] and several other complex trait loci [57-64]. Inheritance of traits and associated variant loci can be tracked genetically using a dense linkage map [65]. Here we examine genome structure using CGH with a custom, 385,585 feature microarray hybridized with genomic DNA from parents and 35 progeny of the cross. We use relative co-hybridized signal intensities between each progeny and the HB3 parent DNA to identify CNVs and to track their inheritance or emergence as *de novo *events within progeny lines. Many CNVs segregated in the expected Mendelian fashion, while a surprising number of CNVs appeared as *de novo *events in one or more progeny clones. Notably, these structural genome variants spanned many genes. We assessed their potential impact on genome-wide transcription, highlighting the likely important role for CNVs in parasite evolution and adaptation.

## Results

### Genome-wide frequency of copy number variants

We investigated genome wide distribution, frequency and characteristics of CNVs within a segregating population of progeny derived from a genetic cross between a multidrug resistant and a generally drug sensitive parasite [56]. We focused on CNVs of approximately 1 kb or larger, with at least 3 probe signals supporting the CNV call.

**Figure 1:**
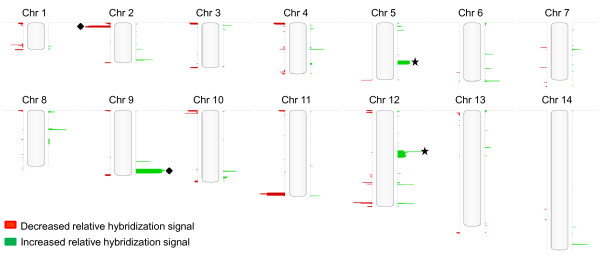
**Genome-wide distribution of CNVs in the progeny of the HB3 × Dd2 genetic cross**. Locations of 170 CNVs in Dd2 and progeny clones compared to the HB3 reference are illustrated across the 14 chromosomes. The length of each bar represents the frequency of the event within the progeny population, and the width of contiguous bars along the length of the chromosome corresponds to the size of the event. Increased relative probe signal intensity is in green, while decreased relative signal is in red. Among the progeny, we observe examples of deletion and amplification events linked to key parasite phenotypes (star - resistance to known antimalarials, diamond - cytoadherence and gametogenesis).

One-hundred and seventy CNVs were detected in at least one parent or progeny clone, affecting 2.5 Mb of the 23 Mb genome and involved 10% of all genes (Table 1). Figure 1A illustrates the genome-wide distribution of CNVs and their frequency in the progeny population. A complete catalogue of the CNVs (position, size, gene content, and number of progeny harboring the CNV) is provided in Additional file 1. Using a stringent CNV calling algorithm [http://www.biodiscovery.com/index/nexus, see methods], we detected 15 of 22 CNVs reported by one group [19] and 3 of 7 reported by another group [20] in the HB3 and Dd2 parent clones (Additional file 2). These CNVs include loci linked to drug resistance (Figure 1A, asterisks): amplifications in *pfmdr1 *(Chr 5) [47] and *gch1 *(Chr12) [19]; cytoadherence and gametogenesis (Figure 1A, diamonds): a deletion on Chr 9 in HB3 [66], a deletion overlapping the KAHRP gene in Dd2 on Chr 2 [48]; and the duplication of a segment on Chr 11 in HB3 [43]. A 1.4 kb deletion on Chr 13 in Dd2 was not detected in any of the progeny. Individual progeny genomes carried a median of 36 CNVs, approximately two CNVs per chromosome, with more gains ($\bar{x}$ = 14) than losses ($\bar{x}$ = 11) (Additional file 3).

**Table 1:** Categories of CNVs detected within the HB3 × Dd2 progeny clone population.

	Segregating	*De novo*	All CNVs
Total number	45	124	169
Total number of bases effected (kb)	999.3	1,518.4	2,519.1
Average size of CNV (kb)	22.7	12.2	14.9
Median size of CNV (kb)	12.9	2.8	4.6
Minimum size (kb)	1,034	918	918
Maximum size (kb)	161.0	134.4	161.0
Number of progeny	3 - 35	1 - 22	1 - 35
Number of CNVs with polymorphic genes	24	58	82
Number of genes within CNVs	170	367	537
Number of polymorphic genes within CNVs	42	145	187

### Categories of CNVs

**Figure 2:**
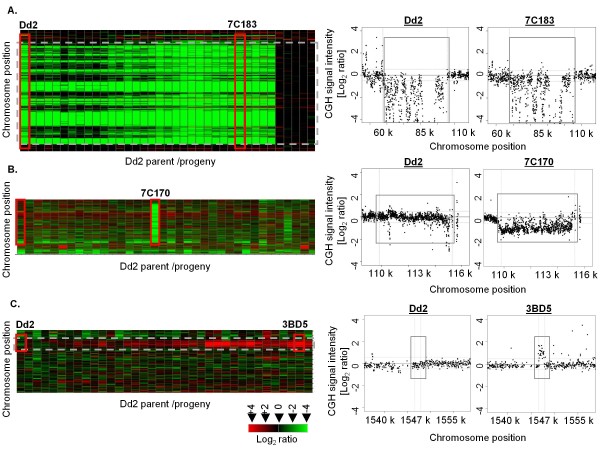
**Categories of CNVs**. Two broad categories of CNVs were identified within the progeny of the HB3 × Dd2 genetic cross: (A) Segregating CNVs are present in a parent clone and are inherited in the progeny, and (B, C) *de novo *CNVs are detected exclusively in the progeny. For each category, the left panel heatmap displays the CNV region (grey boxed) across the Dd2 parent (column 1) as well as the progeny population; the right panel shows a scatter plot of the relative hybridization signal distribution for selected examples highlighted by red boxes. (A) Segregating CNV (deletion) in Chr 2; (B) singleton *de novo *CNV (Chr 4, progeny strain 7C170), (C) recurrent *de novo *CNV detected in Chr 10. In each pair of scatter plots, the left scatter plot shows the signal distribution across the Dd2 parent in comparison with a progeny which carries the CNV (right).

Two major categories of CNVs were defined in the progeny: segregating CNVs were detected in at least one of the parental lines and in at least one of the progeny; CNVs not detected in either parent but observed in one or more progeny were termed *'de novo*'. A *de novo *CNV occurring in a single progeny was sub-designated 'singleton' while *de novo *CNVs which occurred in multiple progeny but in neither parent was sub-designated 'recurrent' *de novo *(Figure 2, Additional file 4 and 5).

Forty-five segregating CNVs ranging from 1 kb to 161 kb affecting 4.3% of the genome (999 kb) and 170 genes were identified (Table 1 and Figure 2A); 42 of these 170 genes were members of polymorphic gene families. In addition to the expected segregating genomic CNVs, 124 *de novo *CNVs were identified (Table 1): 64 singleton (Figure 2B), and 60 recurrent in which the same or similar breakpoints were called in at least 2 progeny (Figure 2B and 2C). Thirty-nine of 60 recurrent *de novo *CNVs were scored in 2 or 3 progeny. Four CNVs were observed in 10 or more progeny and their inheritance pattern indicated that they are probably segregating CNVs (described below).

**Figure 3:**
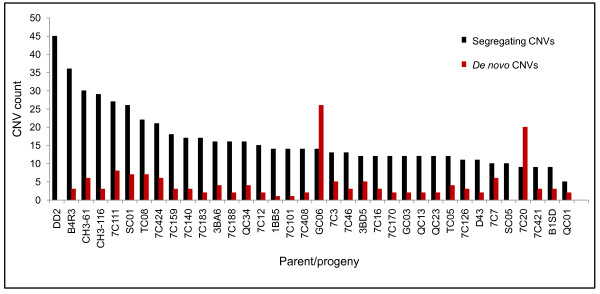
**Total CNVs detected per individual parasite clone**. The number of CNVs per progeny ranged from 8 in QC01 to 45 in B4R3 with a median of 36 CNVs per individual genome. In comparison, the parental genomes contain 45 CNVs. With respect to the CNVs in each individual progeny, segregating CNVs generally constitute the majority, except in GC06 and 7C20 where the majority of the CNVs are *de novo*.

Each progeny gained an average of 4 *de novo *CNVs, including both singleton and recurrent; notably, these events were concentrated in some progeny (e.g.7C20 and GC06), while a single progeny carried none (SC05) (Figure 3). Most *de novo *CNVs (61%) were ≤ 5 kb (Table 1, Additional file 6). Four of the *de novo *CNVs were > 50 kb: a 125 kb amplification in progeny clone 7C20 on Chr 13 (41 genes); a 134 kb amplification in TC05 (41 genes) on Chr13; a 134 kb amplification in progeny clone 7C111 on Chr 8 (39 genes); and a 55 kb deletion in 7C170 on Chr 4 (14 genes) (Figure 2B). Approximately 6.6% of the genome (367 genes) was affected by *de novo *CNVs. Of the recurrent *de novo *CNVs, 55% involved genome regions containing polymorphic genes. Given these three classes of CNVs, we investigated the functional categories of genes that were enriched within the different classes. The most significant (*p *< 0.00005) enrichments are reported in Additional file 7. Genes implicated in drug response, fat metabolism, cytochrome c-heme linkage, aromatic compound biosynthetic process and regulation of DNA replication were enriched in segregating CNVs. Carbohydrate metabolism, meiotic recombination and gamete production were detected as highly significant within the *de novo *CNVs. In all categories of CNVs, pathogenesis, rosetting, cell-cell adhesion, cytoadherence to microvasculature and antigenic variation were enriched, as expected, due to preponderance of polymorphic gene families among the CNV regions.

### CNV Chromosomal locations

**Figure 4:**
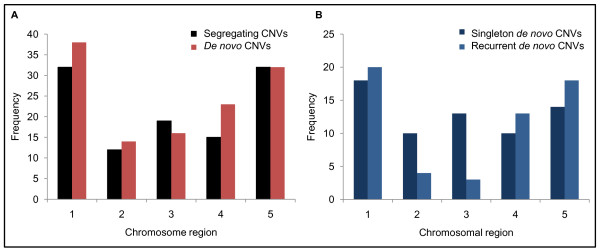
**Chromosomal location of CNVs**. All CNVs detected were placed into 5 chromosomal regions to identify the propensity for localization of CNVs in specific regions of the chromosome. (A) The segregating CNVs were predominantly located in the telomeric/subtelomeric regions. (B) Among the *de novo *CNVs, the singleton CNVs showed a chromosome wide distribution, compared to the telomeric/subtelomeric distribution of recurrent *de novo *CNVs.

CNVs were detected across all 14 chromosomes, spanning 2.5 Mb (11%) of the genome and overlapping 537 genes. For distributional analysis, chromosomes were divided into 5 equal segments and regions were assessed for any biases in CNV counts and categories (Figure 4). Segregating CNVs were observed more frequently in the distal chromosome segments (subtelomeres and telomeres), than were *de novo *CNVs (71% vs 56%) (Figure 4A). Singleton *de-novo *CNVs were distributed chromosome-wide and did not show a regional bias (Figure 4B).

Previous studies proposed amplification/deamplification hotspots [20,67] and fragile genomic regions [44] in *P. falciparum*. We evaluated this possibility by examining distribution of the CNV boundaries in our dataset, assuming a random distribution model. A 10 kb non-overlapping window analysis was used to scan the genome-wide distributions of all 340 breakpoints (each boundary of 170 CNVs). Under random expectation, 3 or more breakpoints within a 10 kb region was highly significant (Poisson model; *p *= 0.00001). This analysis revealed 9 candidate hotspots for CNV breakpoints: one each in Chrs 2, 4, 5, 11, 13, and two each in Chrs 3 and 12 (Additional file 8). All candidate hotspots coincided with regions containing polymorphic gene family members (*PfEMP1, rifin, stevor*, PHIST, DnaJ domain encoding, and cytoadherence linked asexual protein genes). Given that all hotspots were detected in the telomeric/subtelomeric regions, we also looked specifically for hotspots in other regions of the genome. We did not identify additional candidate hotspots in the non-telomeric/subtelomeric regions at high stringency, but did observe 70 positions with two or more breakpoints per 10 kb (*p *= 0.0047).

### Linkage and inheritance of CNVs

A population of segregating sibling parasite clones provides a unique opportunity to track the inheritance patterns of amplifications and deletions. We examined CNVs for Mendelian inheritance, in which case the CNV would be expected to behave as any genetic marker by being inherited in approximately half the progeny clones along with the local allele of its parent of origin, i.e. statistically linked to neighboring markers and mapping to that unique genome location. Using the microsatellite (MS) linkage map [65] CNVs were evaluated for co-inheritance with known markers throughout the genome. In addition, we used the relative hybridization signals of each CNV as a phenotype for quantitative trait loci (QTL) mapping (see methods for details). All 45 segregating CNVs were detected at a minimum score of LOD 2 (logarithm of odds), localizing each to its expected parental allele segment. Twenty-seven of 45 segregating CNV display a highly significant *cis *QTL signal (LOD ≥ 5) mapping to a nearby MS marker (Additional file 9 illustrates *cis *QTL signals for a deletion on Chr 2 and an amplification in Chr 5). Furthermore, by scoring CNVs in the context of their linkage relationships we were able to discover complex subclasses of CNVs (Additional file 100 Additional file 11). Closer examination of the segregating CNVs that were detected only at the lower significance threshold (LOD < 5) revealed several reasons for weaker signal: CNVs with highly skewed inheritance in the progeny population (e.g. Chr 9 [68] - Additional file 10B and Chr 11 [44]); loci with overlapping or neighboring CNVs in the parents (Additional file 10A-i); and complex multiallelic CNVs, i.e. region overlapping a mixture of amplified as well as deleted regions in the parent genomes or *de novo *CNV region overlapping a segregating CNV region in at least a single progeny (Additional file 11B and 11C).

### Inferring mechanisms and CNV origins

**Figure 5:**
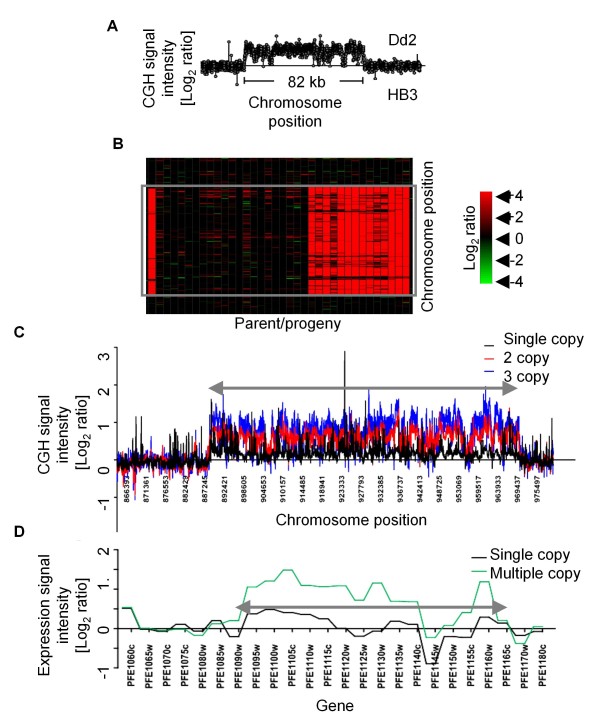
**Non-parent copy number forms at a segregating CNV locus**. We observed non-parental copy number due to amplification/deamplification at the segregating CNV region in Chr 5, which overlaps the multi-drug resistance gene, *pfmdr1*. The size and boundaries of the CNV region of the non-parent form remained identical to that of the parent form indicating that all genes within the amplification were amplified or deamplified. (A) Scatter plot of signal intensity ratios for the Dd2 parent (Dd2vs.HB3) hybridization across an 82 kb segregating amplification highlight the presence of the CNV in the Dd2 parent. Fourteen progeny inherit the amplification. (B) Heat map illustrates increased relative probe signal intensities in Dd2 and progeny lines (red) to the reference HB3 parent (amplified region is marked by a grey box). (C) The scatter plot highlights the relative hybridization signal intensities represented as log_2 _(test/HB3) (amplified region is marked by grey arrow). (C) The progeny exhibit a range of copy number across the amplicon including parent copy number forms as well as non-parent copy numbers reflected by the 3 different groups in the height of the CGH signal intensity across the amplicon. (D) The CNV in the region results in an increase in gene expression (at~18 hrs within the parasite life cycle) in all the multicopy parasites, including the non-parent copy numbers.

**Figure 6:**
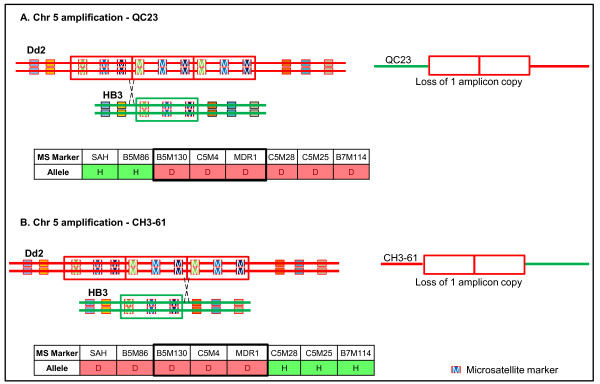
**Role of homologous recombination (HR) in copy number fluctuation in the Chr 5 amplification**. Linkage analysis of the CNV region revealed that of the progeny strains that exhibited copy number fluctuation at the Chr 5 locus, two CNVs were generated by HR between the two parental homologs. The predicted HR patterns and allele distributions in each progeny line A) QC23 and B) CH3-61, are shown with the associated MS marker. The region of the amplicon is highlighted by a black box, and the three MS markers that overlap with the CNV region are shown within the boxed region. D = Dd2 allele, H = HB3 allele.

**Figure 7:**
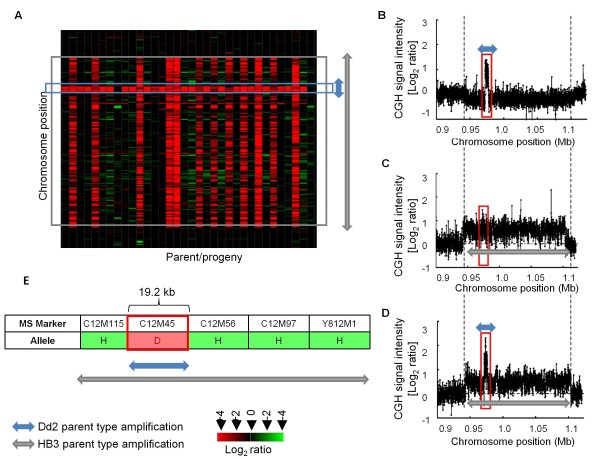
**Inheritance of a complex, multiallelic CNV at a segregating CNV locus**. The Chr 12 locus harbors two unique CNVs: one specific to the HB3 parent (~161 kb, grey arrow) and the other to the Dd2 parent (~5 kb, blue arrow). 34 genes overlap with the HB3-type CNV and 3 genes with the Dd2-type CNV (which are common to both of the CNV types, demarcated by blue box). A) Heat map illustrates the increased relative probe signal intensities in the Dd2/progeny lines (red) in comparison to the reference HB3 parent. (B - D) Scatter plots represent the relative hybridization intensities as a log_2 _(test/HB3) for progeny inheriting Dd2-type CNV allele (B, inherited by 23 progeny) HB3-type CNV allele (C, inherited by 11 progeny) and a single progeny, that inherited both parental CNV alleles at this locus (D). (E) Linkage analysis with MS markers confirms a multiallelic region comprising an approximately 19 kb Dd2 allelic region interspersed within a larger HB3 allelic region overlapping the complex CNV in the progeny strain CH3-61.

**Figure 8:**
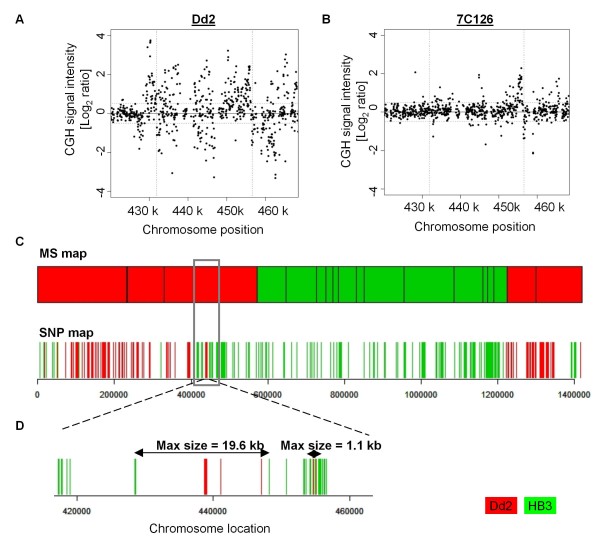
**Inheritance of a *de novo *CNV from gene conversion**. A *de novo *CNV was detected in progeny clone 7C126 in Chr 8. We scrutinized the *de novo *CNV region using MS and SNP allele profiles to assess any allele changes that suggest a meiotic origin. (A, B) Scatter plots representing the relative log_2 _(test/HB3) hybridization intensities for probes representing the *de novo *CNV locus in (A) the Dd2 parent compared with HB3 parent and (B) complex *de novo *CNV in the progeny clone 7C126. (C) Linkage analysis with high density SNP markers [69] of Chr 8 reveals a multiallelic region overlapping the *de novo *CNV region. (D) The two Dd2 allelic regions interspersed within a larger HB3 allelic region was undetected at the lower marker density of the MS map [65] (grey box). The allele profile revealed by the SNP map confirms the meiotic origin of the CNV through gene conversion/double crossover. Each bar of the SNP map denotes a single SNP allele demarcating the parent allele. In both the MS and SNP map the parent alleles are highlighted by red (Dd2) and green (HB3).

To assess possible mechanisms that generate CNVs and their origins, we examined the parental MS inheritance in the regions of both segregating and recurrent *de novo *CNV loci across the progeny. We found no evidence for divergence from Mendelian expectation for segregating CNVs (*p *= 0.99; Additional file 10), simply showing that segregating CNVs tended to be inherited within their expected allele context, i.e. neighboring markers from the same parent of origin. Two of 45 segregating CNVs were perfectly co-inherited with the nearby MS. On the other hand, although strong association with the genotype was evident for the remaining segregating CNVs, it was not perfectly so, with at least a single progeny displaying an allele change in overlapping or neighboring region due to a crossover(s) between the CNV locus and the nearest MS, or due to a local gene conversion overlapping the CNV region detectable only at fine-scale resolution as demonstrated by the examples described below (Figures 5, 6, 7 and 8; Additional file 12).

We inferred from local allele inheritance patterns that several CNVs in the progeny were generated as complex products of recombination. Two segregating CNVs previously implicated in parasite drug resistance, on Chrs 5 and 12, were mapped to their expected reference genome position. However, in the case of the CNV overlapping the Chr 5 *pfmdr1 *locus, not all progeny inheriting the Dd2 *pfmdr1 *allele carry the same number of copies as the Dd2 parent (Figure 5). Of 15 progeny inheriting the Dd2 *pfmdr1 *allele, only 2 have the same 3 copies as the parent; most (87%) progeny with the Dd2 allele have lost at least one copy (4 have a single copy and 9 have 2 copies). One progeny with the HB3 allelic background gained a copy of this locus. In two progeny it could be determined from the parental MS markers allele inheritance pattern that a copy was lost during homologous recombination in meiosis (Figure 6). However, most progeny did not display complex recombination products at this locus that would confirm a meiotic homologous recombination origin. It is probable that in the absence of homologous allele exchange, sister chromatid exchange in mitosis or meiosis could have generated the changes in copy number.

The Chr 12 amplification carrying the *gch1 *locus also demonstrated a complex inheritance pattern in the progeny. Each parent carries a different version of an amplified locus (Figure 7A): the Dd2 parent harbors a ~5 kb amplicon (Figure 7B), while HB3 harbors a ~161 kb amplicon (Figure 7C). All progeny were amplified at this locus, and one progeny clone, CH3-61, uniquely inherited a mixture of the different parental CNVs (Figure 7D). Linkage analysis of the CNV region in CH3-61 shows that a broad HB3 genome segment surrounds a small Dd2 allelic segment, indicating that either a double crossover or gene conversion could have generated this segment (Figure 7E). Given the genome-wide recombination rate (17 kb/cM, [65]) and the size of the physical genome segment affected (maximum distance between nearest markers = 19.2 kb), gene conversion is more likely than a double crossover.

As demonstrated for the recombination products of the *pfmdr1 *and *gch1 *locus, in some cases it is possible to demonstrate meiotic origin by examining the distribution of allelic genetic markers across the genome region of the CNV for its parental origins. Such diagnostic genetic markers require that the parent lines differ for the particular genomic region and that a mapped MS is present in that region, which is often not the case given the genome-wide MS density of 1 marker per 25.5 kb. When parental alleles are not distinct, it is not possible to distinguish the specific type of recombination event that led to the CNV change. Higher marker density provides the resolution to observe local genetic exchange that results in CNV. To investigate the origin of *de novo *CNVs in meiosis, we checked all *de novo *CNVs for their underlying allelic inheritance using the genotype information in the published linkage map [65]. To improve the resolution to 1 marker per 3 kb, we also used our recently published SNP allele dataset derived from sequencing the progeny clone 7C126 [69] to search for evidence of homologous crossover or gene conversion at regions of *de novo *CNV. With this high SNP allele resolution analysis, we characterized two examples of *de novo *CNVs (Figure 8, Additional file 12) in 7C126, and confirmed gene conversion as one potential mechanism by which *de novo *CNVs are generated. The elucidation of precise mechanism(s) will require sequence analysis at CNV breakpoints. For example, whole genome sequencing can systematically identify CNV breakpoints and determine the source of the template for the repair and resolution of genetic exchange events.

Given the large fraction of recurrent CNVs, we examined these more closely to confirm this classification. At the resolution revealed by CGH, exact breakpoints cannot be determined. Consequently, we considered various ways recurrent CNVs can be present; for example some of these may be segregating CNVs that were not detected in the parent CGH. We checked the hybridization signal profiles of all recurrent CNV regions in the parents and assessed all previous work in the parents for CNVs which were not detected in our data but were detected in previously published work that used a range of microarray platforms and probe densities [21-23]. Using this approach we identified 22 *de novo *CNVs that upon visual inspection exhibited characteristics of segregating CNVs. They were missed by our CNV calling algorithm because of their complex nature: for example, presence of overlapping or closely neighboring CNVs in both parents in the CNV region (Additional file 10C). These loci are detected as *de novo *CNVs by the CNV calling software due to variation in hybridization signal in the progeny. In seven of the recurrent CNVs, progeny inherited a mixture of a *de novo *CNV adjoining a segregating CNV (Additional file 11B and 11C), and therefore was classified as a *de novo *CNV.

Recurrent mutations could also occur from low-level subclones within the parent lines used to generate gametes for the cross. This was tested by assessing whether certain *de novo *CNVs co-occur in specific progeny lines reflecting the simultaneous introgression of several CNVs in association with their underlying genetic markers. We did not observe any examples of simultaneous introgression of a subset of *de novo *CNVs that would indicate co-inheritance from a parent subclone. In 37/60 of recurrent *de novo *CNVs, surrounding segments from each parental genome was detected among the progeny with CNV, indicating independent origins (Additional file 11). Twenty-three of the 60 recurrent CNVs were in the context of a single parental genome segment, suggesting either: 1) the CNV is actually a segregating CNV that was missed (or lost) in one of the parent lines; 2) a subclone exists in the parent population that carries the particular CNV and thus 'partially' segregates; or 3) the particular genome segment specific to one parent is a hotspot for *de novo *CNVs. It is important to note that for all 23 cases at least one progeny clone inheriting that parent genome segment did *not *carry the CNV.

The emergence of CNVs in the asexual phase of the parasite life cycle establishes that CNVs can be generated during mitosis in *P. falciparum *[70]. To assess if some of the *de novo *CNVs could have occurred during culture adaptation or cloning during the generation of the genetic cross, we compared genes in *de novo *CNVs with those previously reported from field isolates, laboratory adapted lines or culture adapted lines (Additional file 13). We observed 68 genes in common with previous studies. Incidentally we do not observe *Rh1*, commonly observed to emerge during culture adaptation. We note membrane protein genes (*PfEMP1, Pfmc-2TM*), duffy binding-like merozoite surface protein gene, *Plasmodium *exported protein genes (*PHIST*), an ABC transporter (putative), hexose transporter, DNA/RNA-binding protein Alba (putative), *Gbph2, histidine-rich protein (hrp) iii*, antigen proteins (acyl-CoA ligase antigen, S-antigen) and members of polymorphic gene families (*rifin, stevor, surfin*) among the genes that are common with the *de novo *CNVs.

We also explored the use of QTL to map mechanisms that regulate copy number in the progeny of the genetic cross. This approach used the CNVs as traits with the expectation that QTL loci can reveal gene variants that influence the tendency for different progeny to generate CNVs. For this analysis, we considered *de novo *amplifications and deletions, calculated as a percentage of the total number of events per progeny as distinct phenotypes. We did not detect any QTL loci at the lowest threshold associated with *de novo *amplifications. However, for *de novo *deletions we detected a suggestive QTL on Chr12 (34.3 cM, LOD = 2.39). The locus includes a putative transcription factor Tfb2 (PFL2125c), a subunit of transcription/DNA repair factor TFIIH, that has been implicated in DNA damage response, nucleotide excision repair [71] and chromosome fragility [72].

### Segregation distortion of CNV regions

More than half of the segregating CNVs were inherited in the expected 1:1 Mendelian ratio among the progeny. Segregation distortion was observed for 20 of the 45 segregating CNVs (*p *< 0.05). This included 6 CNVs (4 deletions and 2 amplifications) that were highly skewed: 1) Chr 2 sub-telomeric deletion of the *kharp *(PFB0100c) locus, deleted in Dd2 and 86% of progeny; 2) Chr 9 sub-telomeric locus, deleted in HB3 and all progeny; 3) Chr 12 sub-telomeric locus, deleted in HB3 and 77% of progeny; 4) Chr 13 locus, deleted in HB3 and 91% of the progeny); 5) Chr 11 sub-telomeric CNV, amplified in HB3 and 86% of the progeny; and 6) Chr 12 amplification of the *gch1 *locus, amplified in both parents, and higher copy number than HB3 in 97% of the progeny, at the *gch1 *locus (see Figure 7A). Five of these agree with the previously reported regions of segregation disparity proposed to reflect the survival advantage of favored haplotypes during the generation of the HB3 × Dd2 cross [73].

### Impact of CNVs on gene expression

We integrated a previously generated gene expression data set for the HB3 × Dd2 genetic cross with the current CGH data to assess the impact of CNV on gene expression. QTL mapping of transcript abundances as quantitative traits identified both local regulatory effects (e.g. *cis*-regulation) and distant effects (*trans*-regulation) [74]. Both segregating and *de novo *CNVs showed an impact on the expression of resident genes (Additional file 14). Of the 539 genes impacted by CNVs, 170 resided in segregating CNV. These CNVs extensively influenced the inherited levels of transcription of the genes residing within the CNV (Figure 5D), as well as distant (unlinked) genes, than would be expected by chance. For example, 77 of the genes residing in 8 segregating CNVs were differentially regulated locally, indicating strong local regulation due to altered gene dosage. An additional 353 genes scattered throughout the genome were regulated in *trans *by loci that coincided with segregating CNVs. This implies that a gene(s) residing in the CNV has an effect on downstream transcripts either directly as a regulatory protein, or indirectly through physiological or signaling role. Amplifications were the predominant CNV that influenced transcription via both *cis *and *trans *mechanisms. Several loci influenced the expression of a large number of genes, and were identified as regulatory hotspots [74]. Five of the 12 eQTL hotspots aligned with segregating CNVs: three in Chr 5, one in Chr 7 and one in Chr 12. One of the hotspots in Chr 5 (65.9 cM) and one in Chr 12 (103.3 cM) correspond to amplifications implicated in resistance to known antimalarial drugs.

## Discussion

Recent studies of *P. falciparum *demonstrated the widespread prevalence of CNVs in populations and their likely adaptive influence on important traits such as drug resistance [75,76]. Large scale amplification and deletions have been known for several decades [39-42]. However, a precise understanding of genome plasticity, origins of CNVs and their stability, including transient and reversible fluctuations in a generational time-frame is deficient not only for the malaria parasite, but for other organisms as well [49]. For example, little is known about the behavior of copy number variant regions, the rate of reversion to an original state, the rate at which new variants arise, and the uniformity of the distribution of new variants in a sibling population. The segregating population examined in this study provides an ideal context in which to view the inheritance and stability, and occasionally to infer the origin of a CNV. We report extensive plasticity and segregation complexity of CNVs within the progeny.

Three different classes of CNVs - segregating, singleton *de novo *and recurrent *de novo *- were prominent in this study and are contrasted here for their inheritance patterns among progeny clones (Table 1, Figure 2, Additional files 4 and 5). Among these three classes, we observed duplications, deletions and multiallelic complex loci, as has been described for CNVs in human [25,49] and chicken [14] (Additional files 4, 5 and Figure 7). We observed many *de novo *CNVs (Table 1). Information on *de novo *CNVs has been scarce because previous studies did not examine parent-progeny populations. With the availability of suitable genetic systems along with high-throughput technologies which enable genome wide discovery of CNVs, it is clear that *de novo *CNVs are an important source of genetic variation [49,53,77]. Furthermore, *de novo *events are not unprecedented in *P. falciparum*. Duplication of subtelomeric sequence has been documented previously in progeny of different genetic crosses including the HB3 × HB3 self cross [42]. Previous development of the MS linkage map revealed non-canonical MS markers in the HB3 × Dd2 [65] and non-parental sequence products in the HB3 × 3D7 [78] as well as the HB3 × Dd2 [43] genetic crosses, further emphasizing the genome plasticity of the parasite both at smaller (< 1 kb) as well as larger (> 1 kb) scales of sequence.

Our data provide clear evidence for copy number differences from the parent lines within the segregating progeny population. Most of the previously known segregating CNVs exhibited a Mendelian segregation pattern at a broad scale and mapped to markers close to their genome positions (Additional file 9). However, finer scale scrutiny of two segregating CNVs implicated in drug resistance revealed unique structural changes resulting from meiotic recombination events. The Chr 5 *Pfmdr1 *amplification which has been associated with Mefloquine resistance [79,80] and is widely detected in natural parasite populations [76], exhibited both loss and gain of copies compared to the parental state (Figure 5). This highlights that both amplification and deamplification mechanisms have affected the locus. Similarly, the *gch1 *locus, postulated to be associated with antifolate resistance [81] and widely detected in parasite populations [75], also exhibited complex multiallelic copy number within a single meiotic generation (Figure 7). These examples illustrate the highly dynamic nature of CNV regions during a single meiotic generation that would not be recognized in a standard population-based CNV survey.

Four mechanisms can generate CNVs and lead to fluctuation of copy number in the CNV regions: homologous recombination (HR), non-allelic homologous recombination (NAHR), non-homologous end joining (NHEJ) and the replication based mechanism, microhomology-mediated break-induced replication (MMBIR) - which includes Fork Stalling and Template Switching (FosTes) [82]. The absence of factors in the malaria parasite genome required for NHEJ combined with evidence for HR and NAHR from both laboratory genetic crosses and field isolates argue that recombination mechanisms play a central role in generating genetic diversity in the parasite. Consistent with previous reports, we demonstrate that recombination generates amplifications and deamplifications of both segregating and *de novo *CNVs. We show evidence of recombination detected by local allelic changes that resulted in copy number loss (Figure 6) and gain (Figure 6 and 7) in segregating CNVs and gain of *de novo *CNV (Figure 8, Additional file 12). While Chr 5 CNVs in two progeny clearly indicate HR origins, lack of evidence for reciprocal allele exchange in other progeny implies that most CNVs may appear due to unequal HR between sister chromatids. Unequal sister chromatid exchange is postulated as a mechanism that generated the multiple independent events of the *pfmdr1 *CNVs within parasite isolates [47]. MS allelic changes at the Chr 12 locus (*gch1*) in our data indicate copy number fluctuation by sister chromatid exchange, a double crossover or gene conversion. Gene conversion has been reported to generate diversity within multigene families in *P. falciparum *[42]. Duplication of chromosomal segments by gene conversion, including duplicative translocation, has been described in genetic crosses [42] and parasite clones [83]. Alternatively, complex multiallelic/mosaic regions can result from gene conversion which can change the CN profile from that of the parents [83], an observation consistent with the several examples of *de novo *CNVs described in this study (Figure 8 and Additional file 12).

In general, it is difficult to establish CNV origins. The steps involved in generating a genetic cross include many opportunities for both sexual and asexual (in meiosis and mitosis) genetic exchanges [42,47,78,83]. A more precise inference of mechanisms would benefit from knowledge of the number of mitoses that each parent lineage underwent prior to the generation of gametes for the cross, as well as the number of mitotic replication cycles that the parent and progeny parasites underwent after meiosis. Although allelic marker co-inheritance can pinpoint homologous recombination as one origin of CNVs when sufficient sequence differences can distinguish the parental allele segments, this method cannot differentiate the CNVs generated in asexual replication or in genomic regions that are identical (or very similar) in the parents.

While unlikely, it cannot be ruled out that recurrent mutation reflects parent subclone populations (i.e. gamete mixtures). Although parasites were cloned by micromanipulation or limiting dilution, and it is generally accepted that this method would produce true single-clone parent lines, we are necessarily dealing with these 'individuals' as populations expanded in culture. Therefore, it is possible that genetic changes arising in these cultured lines in preparation of gametes for the cross could contain mixed genotypes that are represented in the gametes which segregate into some subset of progeny clones. We found some recurrent *de novo *CNVs residing in both parent allele backgrounds that suggested independent origin. Furthermore, we did not find evidence for simultaneous introgression of CNV, which should be readily apparent in the presence of two or more distinct parent subclones. Overlap of several single as well as recurrent *de novo *CNVs with CNVs reported to have arisen under culture adaptation and/or *in vitro *culture, suggests that several *de novo *CNV regions may have emerged in culture adaptation (Additional file 13) but cannot be precisely determined at CGH resolution.

We noted a preponderance of CNV breakpoints within narrow genomic regions, including recurrent *de novo *CNVs that impacted the same genome segments. Genomic regions that show a high propensity for segmental duplications also have been suggested in isolates [20] and laboratory lines [22] of *P. falciparum*. Additionally, previous work has also demonstrated extensive occurrence of deletions particularly in the subtelomeric sequences [44,46,48,84,85], indicating that the subtelomeric regions may be highly unstable and represent fragile sites [85,86]. It has been postulated that specific sequence features may underlie the fragility of the subtelomeric regions [85]. Recurrent structural mutation has been observed in mice [49] and humans [53] during inheritance. Similarly, recurrent duplication has been detected previously in *P. falciparum*; especially in association with the subtelomeric regions in progeny of both the HB3 × Dd2 and HB3 × 3D7 genetic crosses [42], while recurrent subtelomeric deletions have been detected in independent clones of a field isolate [85]. Several recent studies have demonstrated recurrent mutations as a key mechanism by which gene copy number fluctuations take place within short generational time scales [49]. These studies have emphasized that recurrent CNVs may be an important biological process in evolution, as well as human disease [7,53].

Skewed inheritance was observed for a majority of the segregating CNVs. Skewed inheritance was expected to an extent, given that skewed inheritance of parental alleles were previously noted within this population for seven regions, mostly located in the sub-telomeres, during construction of the MS linkage map [65]. Consistent with the expectation from MS linkage analysis, five of the CNV regions overlapped with the skewed allele distributed regions in the MS map, emphasizing the role of CNVs in parasite selection. The skewed regions overlap with genes associated with parasite pathogenicity [87], gametogenesis [44,46] and drug resistance [19]. Regions of skewed inheritance have been observed not only in the HB3 × Dd2 genetic cross [42,65], but also in other independent genetic crosses [42,88,89]. It has been suggested that the skewed inheritance may be related to the selection of alleles beneficial for parasite viability, growth and proliferation in a splenectomized chimp during the generation of the genetic cross and/or in parasite growth under *in vitro *growth conditions [65]. If deletions are eliminated by selection, populations that emerge in culture should carry more amplifications than deletions. This trend was observed in the progeny clones carrying more gains than losses (69%, Additional file 3).

The stability and fitness of CNV loci is postulated to play an important role due to their implication in resistance to antimalarials [67]. Previous work supported a co-adaptive role of *pfmdr1 *copy number with the CQ resistance gene *pfcrt*. Inheritance of these loci in the progeny clones of the HB3 × Dd2 has suggested an influence on fitness due to the presence of specific combinations of alleles that exist among the progeny. It was observed that high *pfmdr1 *copy number is maintained only in the context of its co-selected mutant *pfcrt *partner and CQ sensitive *pfcrt *is never paired with 3 copies of *pfmdr1 *[63]. Two groups indirectly evaluated the *in vitro *dynamics and possible fitness effects of CNV in *P. falciparum *[67,90]. Both attempted to address the fitness effects at a single CNV locus, in the presence and absence of drug pressure, using a single strain of *P. falciparum*. Each proposed a fitness cost associated with carrying the multicopy CNV as indicated by the out-growing of the single copy over the multicopy parasite in a mixture of parasites. Mathematical modeling of *in vitro *based experimental data suggested a CNV emergence rate of 1 in 10^8 ^parasites [67]. The rate of emergence in the population is ultimately a reflection of the rate of de-amplification as well as parasite growth dynamics due to fitness costs associated with carrying higher copy numbers.

Emergence of CNV under *in vitro *conditions have been reported widely in *P. falciparum *with laboratory adaptation [68,91,92], under long term laboratory culture [19-22] and under drug pressure [21,23,67,90,93]. It has been widely postulated that parasites have fewer constraints during *in vitro *culture conditions such that growth advantages can be gained from decreased investment in activities such as protein exportation, knob construction, display of cytoadhesive molecules and variant antigens, and production of gametocytes [24]. The overlap we observed of *de novo *CNVs with some of these genes is consistent with the interpretation that culture adaptation and cloning could be associated with lost functions via deletions.

Along with extensive chromosomal size variation identified previously by PFGE [38,42,43], our data demonstrate a highly plastic genome with strong potential to influence function through gene dosage effects. We explored the potential functional impact of CNVs. Functional enrichment analysis of the *de novo *CNVs revealed genes involved in carbohydrate metabolism, recombination and gametogenesis; while segregating CNVs involved drug response, fat metabolism, aromatic compound biosynthetic processes and regulation of DNA replication in *P. falciparum*. In both segregating and *de novo *CNVs, functions of polymorphic gene families were represented. The presence of functional gene families has been taken as an indication of positive selection on gene duplications over time [25,94]. Gene duplication is now recognized as an important mechanism for evolution of new biological functions in organisms [94]. CNVs in humans are enriched for genes involved in molecular interactions to specific environmental stimuli including drug detoxification, immune response, cell surface integrity and surface antigens. It has also been postulated that CNVs could carry genes that contribute to inter-individual variation and can play a role in the differences in drug response and immune defense [27], but not in intracellular processes such as biosynthetic and metabolic pathways [95]. The genome wide distribution of CNVs and the abundance and breadth of genes overlapping CNV regions, as well as their widespread involvement in local and distant gene regulation, indicate the extensive contribution of CNVs in phenotypic variation, similar to that observed in human studies [25].

## Conclusions

We describe the breadth and distribution of genome-wide CNVs detected in a segregating parasite population and a more dynamic genome structure than has been reported previously for malaria parasite populations. We highlight CNVs arising *de novo *in the progeny clones. The classical genetic framework provided a unique opportunity to examine the Mendelian behavior of CNV regions, including the identification of allele segregation patterns that indicate mechanisms that generate CNVs. We also directly tested the impact of CNVs on gene expression by overlaying eQTL and report widespread effects of local and distant regulation. By using a segregating genetic system to study the breadth, distribution and dynamics of CNVs, we reveal an extremely plastic parasite genome in which CNVs are a prominent source of diversity and maybe an overlooked substrate for selection.

## Methods

### Parasite culturing and DNA isolation

Parents and progeny of the HB3 × Dd2 genetic cross were obtained from the original cloned stocks. The HB3 × Dd2 genetic cross consists of 35 haploid progeny, mimicking, in effect, recombinant inbred lines for linkage analysis. Each progeny was previously genotyped for 901 restriction fragment length (RFLP) and MS markers spanning the 14 chromosomes (~23 Mb) at a resolution of one crossover every 40 kb [65]. All parasites used in this experiment were cultured in human erythrocytes (RBCs) by standard methods [96,97] utilizing leukocyte-free human RBCs (Indiana Regional Blood Center, Indianapolis, Indiana) suspended in complete medium (CM) [RPMI 1640 with L-glutamine (Invitrogen Corp.), 50 mg/L hypoxanthine (Sigma-Aldrich), 25 mM HEPES (Cal Biochem), 0.5% Albumax II (Invitrogen Corp.), 10 mg/L gentamicin (Invitrogen Corp.) and 0.225% NaHCO3 (Biosource)] at 5% hematocrit. Cultures were maintained independently in sealed flasks at 37°C under an atmosphere of 5% CO_2_, 5% O_2_, and 90% N_2_. Parasitemias were monitored and generally maintained at 5-7%. DNA was extracted from each parasite culture using standard phenol/chloroform protocols and concentrated using salt precipitation for labeling and hybridizing to CGH microarrays.

### Comparative genome hybridizations

A high resolution CGH microarray, designed with 385,585 probes representing the entire *P. falciparum *3D7 reference genome by NimbleGen Systems, Inc. (Madison, Wisconsin) was used [98]. Probes were isothermally designed (Tm-balanced) and adjusted in length to maintain an optimal fixed hybridization temperature. Probes are on average 56 bp in length and spaced at a median of 21 bp across the genome. Probes overlapped at a median of 31 bp with 58.3% of the probes having some overlap. The remaining had either no overlap (1.4%) or gaps between probes (40.3%). Probe coverage density and the frequency of probe overlap were dependent on the complexity of the DNA sequence. Regions with long tracts of repetitive DNA are not well represented on the microarray and resulted in probe gaps.

Genomic DNA (gDNA) from the 35 progeny and the Dd2 parent parasite line were co-hybridized to CGH microarrays with the parent line HB3 as a common reference using the standard NimbleGen CGH protocol [99]. Briefly, genomic DNA fragmentation, labeling, hybridization, washing, and scanning were carried out using the standard NimbleGen CGH protocol at the NimbleGen Service Laboratory. For each spot on the microarray, log_2 _(Cy3/Cy5) were calculated for Cy3 and Cy5 labeled test and reference samples, respectively. Normalization of the Cy3/Cy5 signal was performed for each microarray using the Qspline algorithm (normalize.qspline, http://www.bioconductor.org).

### Data visualization

Each probe was blasted (NCBI BLAST 2.1.1, without low complexity filtering) against the 3D7 *Plasmodium falciparum *reference genome (PlasmoDB v5.4, [100]) and non-unique probes were discarded. A total of 383,333 probes were used for CNV analysis. The microarray data were visualized via scatter plots and heat maps using Spotfire DecisionSite v8.2 (TIBCO Spotfire; Somerville, Massachusetts) and R language [101].

### CNV detection criteria

The filtered set of unique probes was used for CNV detection. Segmentation analysis for identification of CNV regions and further visualization was performed using Nexus Copy Number 3.0 software (BioDiscovery, Inc.; El Segundo, California). The CNV detection was performed using the rank segmentation algorithm of Nexus with significance threshold of 1.0E-10 and a Max Contiguous Probe Spacing (Kbp) of 1000. Because *P. falciparum *is a haploid organism, relatively low single value cutoffs of log_2_ratio of normalized Cy3/Cy5 values of 0.5 and -0.5 were used to call CNVs. Additionally for a region to be considered a CNV, we required the region to carry three or more probes, and the distribution of the log_2_ratio value of the normalized Cy3/Cy5 values of all the probes spanning a CNV region was compared to the normalized Cy3/Cy5 values of a selected set of probes that are known to be non-polymorphic in both parental and the 3D7 genomes. The skewedness of the signal distribution across the CNV regions was compared against the expected normal distribution of a non-CNV region (mean = median = log_2_ratio = 0).

As the reference genome 3D7 was used for the design of the probe sequences, sequence segments present uniquely in the parental genomes which are absent in 3D7 will be unrepresented in the array design. Therefore CNVs which may overlap with these regions will remain undetected in this study. Although the semi-tiled array design used in this study enables large scale detection of most of the CNV regions, due to the highly repetitive nature of the parasite genome, certain regions which contain no or very low probe density will also remain undetected. Thirdly, the CNVs were identified by comparison to parental genome HB3. Segments amplified in HB3 will appear as losses in the test samples, or may be completely missed as CNV regions if both parental genomes carry it and is inherited in the progeny.

### Quantitative PCR (qPCR)

Quantitative PCR was carried out with SYBR green PCR Master Mix (Applied Biosystems) using an ABI 7900HT sequence Detection System. For selected CNVs, primers were designed using Primer Design software (ABI) with standard parameters in each gene spanning the CNV region as well as two genes outside the region. For each primer pair, 4 reactions were set up for the test DNA, and the reference DNA. For quantification and comparison across samples, each qPCR plate included a control locus (*beta tubulin *gene) known to be a single copy gene in both the test and the reference sample. Relative copy number was calculated using the ΔΔC_T _method.

### GO enrichment analysis

GO enrichment for genes within the different categories of CNVs was calculated using MADIBA [102], a web source for biological analysis of *Plasmodium *genes. *Plasmodium falciparum *genome 2007 release was used for enrichment analysis. The *p*-value is calculated using a hypergeometric test which determines if the number of times that a GO term appears in the cluster is significant, relative to its occurrence in the genome. The result is significant if the *p*-value is less than 0.05 (at a 95% confidence level) [102].

### Identification of regional and location biases of CNVs

Each chromosome was divided into 5 equal regions. The frequency of segregating, singleton *de novo *and recurrent *de novo *CNVs observed in each regions was calculated to identify regional biases in CNV distribution. A non-overlapping 10 kb chromosome-wide window analysis was used to investigate 'hotspots' of CNV using the breakpoints of all CNVs (170 CNVs, 340 unique breakpoints). A random Poisson model was used to locate significant windows of CNV hotspots (X > 2, λ = 1). To identify other hotspots which may exist in the non-subtelomeric/telomeric regions a finer-level analysis was carried out, given a random Poisson model, after the removal of the telomeric/subtelomeric regions. The telomeric/subtelomeric regions were defined as in Mok et al. [103].

### Investigation of allele identity, linkage and CNV

QTL analysis was performed for log_2 _signal intensity ratios for each probe on a DNA microarray for the progeny of the HB3 × Dd2 (co-hybridized with the HB3 reference DNA). Probes that overlap a DNA polymorphism in the test or reference DNA sample are detected as deviations from the log_2_ratio = 0. If a particular polymorphism segregates among the progeny, QTL associated with the probe will be localized with the respective MS marker position in the linkage map. QTL analysis was carried out by computational approaches described previously [104] using Pseudomarker (Version 2.04, http://churchill.jax.org/software/archive/pseudomarker.shtml). A high significance threshold (LOD ≥ 5) as well as lower LOD thresholds of LOD ≥ 3 and LOD ≥ 2 was used for QTL analysis.

Chi square test was used to test for uniformity in allele identity overlapping CNV regions in segregating as well as recurrent *de novo *CNVs using the MS linkage map [65]. Probe signal overlapping the CNV regions were used as a 'trait' and mapped as a QTL to identify strong segregating CNVs for identification of candidate markers. CNVs deviating from expected observations were investigated individually using scatter plots and heat maps (Spotfire DecisionSite v8.2, and R language [101]).

The mechanisms of copy number change were inferred by investigating the copy number (qPCR) of one or more genes within the CNV with the pattern of allele distribution of MS markers adjoining and overlapping CNV regions. A previously generated high density SNP map for progeny clone 7C126 [69] was used to specifically look for signs of gene conversion or crossover in *de novo *CNV regions to infer mechanism(s) of *de novo *CNV.

To deduce genes that underlie the inherited differences in the machinery that influence the tendency to generate CNVs, copy number was mapped as a trait in QTL mapping. The frequency of *de novo *amplifications and *de novo *deletions were calculated as a percentage of the total number of CNVs per progeny. QTL analysis was carried out by computational approaches described previously.

### Segregation disparities of CNV regions

Skewed inheritance of segregating CNVs were assessed using a Fischer's exact test comparing the observed number of progeny with an event to the expected number of progeny with the same event assuming a 1:1 Mendelian inheritance at each locus in the genome.

### Gene expression and eQTL analysis

A previously generated gene expression data set (at approximately 18 hrs in the life cycle) for the progeny of the HB3 × Dd2 genetic cross [74] was integrated with the current CGH data to assess the impact of CNV on the expression of genes that reside within the event. Similar to the CGH microarrays used here, Dd2 and progeny cDNA samples were co-hybridized with a common reference, HB3 cDNA sample. Gene expression of CNV regions was compared to the expression of non-CNV regions for both segregating as well as *de novo *CNVs, using Welch's t-test (*p *< 0.05) [105]. The genome-wide analysis of expression QTL (eQTL) loci and hotspots was integrated to assess the impact of CNVs in gene expression changes that have occurred in the progeny population [74]. Random genome-wide expectation for eQTL was calculated by computing the number of eQTL associated with a random set of 537 genes (the number of genes which overlap CNV regions). An average was calculated under 1000 iterations (*cis *= 23.01 ± 4.4 genes, *trans *= 72.5 ± 7.5 genes), and compared with observed eQTL associated with CNVs. eQTL loci were assessed for segregating CNV regions spanning from 50 kb (~3 cM) upstream to 50 kb (~3 cM) downstream of the CNV breakpoints.

## Authors' contributions

JP and MTF conceived the study. US, JG, JP, AT and MTF performed data analysis. US performed the qPCR, MS genotyping and DNA sequencing. LC grew parasites and obtained DNA for microarray experiments. US, JG, and MTF wrote the paper. All authors have read and approved the final manuscript.

## Supplementary Material

Additional file 1 **Catalogue of CNVs in the HB3 × Dd2 genetic cross**. Click here for file

Additional file 2 **Known chromosomal polymorphisms detected by CGH in HB3 and Dd2**. Click here for file

Additional file 3 **Loss and gain frequency of CNVs across the progeny**. In general the progeny population shows an accumulation of gains than losses (average gain = 14, average loss = 11). 69% of the progeny have more gains than losses. Click here for file

Additional file 4 **Hybridization signal distribution in segregating and *de novo *amplifications**. The distribution of the log_2_ratio of the progeny hybridization signals at segregating and *de novo *CNV regions were assessed in comparison with that of the parental signal (Dd2/HB3). The positively skewed signal distribution highlights duplicated CNV regions. The clear absence of skewed signal in the Dd2/HB3 parental hybridization compared to that of the positively skewed signal distribution in progeny enabled the identification of *de novo *amplifications. Click here for file

Additional file 5 **Hybridization signal distribution in segregating and *de novo *deletions**. The distribution of the log2ratio of the progeny hybridization signals at segregating and *de novo *CNV regions were assessed in comparison with the parental signal (Dd2/HB3). The negatively skewed signal distribution highlights deleted CNV regions. The clear absence of skewed signal in the Dd2/HB3 parental hybridization compared to that of the negatively skewed signal distribution in the progeny enabled the identification of *de novo *deletions. Click here for file

Additional file 6 **Size distribution of segregating and *de novo *CNVs**. The size distribution of the CNVs was assessed as a percentage of total CNVs in each category. *De novo *CNVs were predominantly < 10 kb (76%), while segregating CNVs were > 10 kb (55%). In both segregating and *de novo *CNVs, a small percentage of CNVs were > 100 kb (segregating = 4%, *de novo* = 2%). Click here for file

Additional file 7 **Gene enrichment within categories of CNVs**. Click here for file

Additional file 8 **Hotspots of CNV breakpoints**. Click here for file

Additional file 9 **Genetic linkage in selected CNV regions**. The relationship between linkage position and genome location was assessed by QTL mapping, using relative hybridization signal per probe in segregating CNV regions as a phenotype. Each individual probe signal of segregating CNVs mapped to its closest MS marker in the published linkage map for the HB3 × Dd2 genetic cross [65], highlighting the colinearity of the linkage and physical genome at the CNV regions. The pattern remained true for progeny wide inheritance of A) amplified regions (e.g. Chr 5, boxed in red) as well as, B) deleted regions (e.g. Chr 2, boxed in red). Click here for file

Additional file 10 **Allele distribution in segregating CNV regions**. We directly examined the parental MS inheritance using the published linkage map for the HB3 × Dd2 genetic cross [65] overlapping the regions of segregating CNVs, in each progeny. (A) The expected number of CNVs was compared to the observed parental allele of the CNV region. We found no evidence for divergence from Mendelian expectation (chi square test, *p* = 0.99). A few CNVs (e.g. i-v) deviated from this expectation due to lack of marker coverage adjacent to the CNV locus and/or complexity of CNV region in parents or progeny, including two regions that has been previously known to display skewed [53] or complex allele distributions: B) single progeny with a complex CNV overlapping a segregating CNV region (A-ii) and C) complex CNV region in parent genomes (A-iv). Selected CNVs are shown by grey boxes within heat maps (Dd2 parent in column 1) and are highlighted by scatter plots. Click here for file

Additional file 11 **Allele distribution in recurrent *de novo *CNVs**. We directly examined the parental MS inheritance [53] adjacent/overlapping the recurrent *de novo *CNVs in progeny. (A) Curiously, most CNVs were observed to carry one parental allele in progeny with the CNV. CNVs which were widely recurrent (> 5 progeny) were investigated closely and were discovered to be: (B) segregating regions (boxed in red) within which one of more progeny exhibited overlapping *de novo *CNV (boxed in gray) and/or (C) segregating complex regions (one or more CNVs in one or both parents). Selected CNVs are shown in boxed regions in the heat maps (Dd2 parent in column 1) and highlighted by the scatter plots. Click here for file

Additional file 12 **Recurrent *de novo *CNV in a multiallelic region**. We directly examined the parental SNP allele inheritance [69] within a recurrent *de novo *CNV in Chr 12 in the progeny clone 7C126. The *de novo *CNV region is demarcated by an arrow (A) scatter plot of parent CNV profile, Dd2 parent is compared with HB3 parent; (B) scatter plot of progeny CNV profile, progeny is compared with HB3 parent. (C) SNP map of Chr 12 [69]. Each bar of the SNP map denotes a single SNP allele demarcated by the parent allele. The parent allele is highlighted by red (Dd2) and green (HB3). The SNP allele profile which overlaps the *de novo *CNV region confirms a HB3 allelic region interspersed within a larger Dd2 allelic region (highlighted by arrow), suggesting a potential gene conversion or double crossover. Click here for file

Additional file 13 ***De novo *CNV genes that overlap with CNVs in laboratory and culture adapted field isolates**. Click here for file

Additional file 14 **Impact of CNVs on gene expression**. A previously generated data set of gene expression at 18 hrs in the HB3 × Dd2 progeny population [74] was assessed for impact of CNVs on gene expression. All categories of CNVs resulted in an impact on the gene expression when compared with the gene expression of progeny that do not show CNV in the respective regions. Click here for file
