# Supplementary material for: The landscape of inherited and de novo copy number variants in a plasmodium falciparum genetic cross
Source: BMC Genomics. 2011 Sep 22;12:457. doi: 10.1186/1471-2164-12-457 (PMC3191341; doi:10.1186/1471-2164-12-457)
Supplement: Additional file 3 — Loss and gain frequency of CNVs across the progeny. In general the progeny population shows an accumulation of gains than losses (average gain = 14, average loss = 11). 69% of the progeny have more gains than losses. [file 1471-2164-12-457-S3.PPTX]

## Slide 1
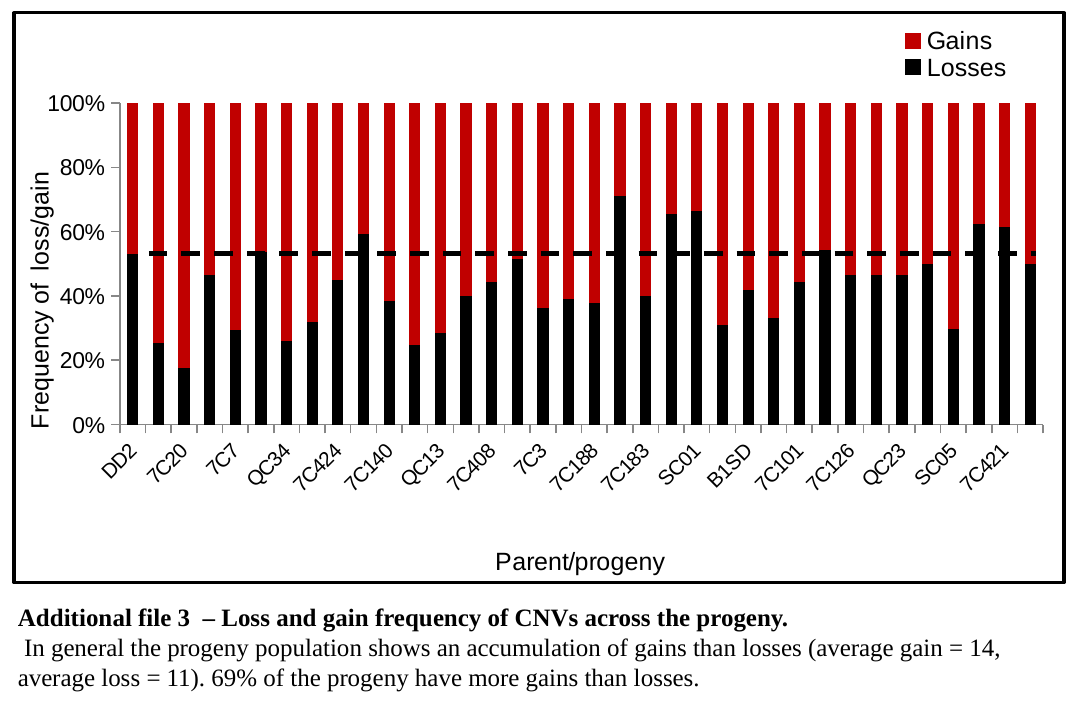

### Chart
| Category | Losses | Gains |
|---|---|---|
| DD2 | 24.0 | 21.0 |
| GC06 | 11.0 | 32.0 |
| 7C20 | 6.0 | 28.0 |
| TC08 | 22.0 | 25.0 |
| 7C7 | 8.0 | 19.0 |
| B4R3 | 22.0 | 19.0 |
| QC34 | 6.0 | 17.0 |
| 3BD5 | 8.0 | 17.0 |
| 7C424 | 14.0 | 17.0 |
| CH3-61 | 25.0 | 17.0 |
| 7C140 | 10.0 | 16.0 |
| 3BA6 | 5.0 | 15.0 |
| QC13 | 6.0 | 15.0 |
| 7C16 | 10.0 | 15.0 |
| 7C408 | 12.0 | 15.0 |
| 7C12 | 16.0 | 15.0 |
| 7C3 | 8.0 | 14.0 |
| 7C159 | 9.0 | 14.0 |
| 7C188 | 8.0 | 13.0 |
| 7C111 | 32.0 | 13.0 |
| 7C183 | 8.0 | 12.0 |
| CH3-116 | 23.0 | 12.0 |
| SC01 | 24.0 | 12.0 |
| GC03 | 5.0 | 11.0 |
| B1SD | 8.0 | 11.0 |
| 7C170 | 5.0 | 10.0 |
| 7C101 | 8.0 | 10.0 |
| TC05 | 12.0 | 10.0 |
| 7C126 | 7.0 | 8.0 |
| D43 | 7.0 | 8.0 |
| QC23 | 7.0 | 8.0 |
| 7C46 | 8.0 | 8.0 |
| SC05 | 3.0 | 7.0 |
| 1BB5 | 10.0 | 6.0 |
| 7C421 | 8.0 | 5.0 |
| QC01 | 4.0 | 4.0 |Additional file 3 – Loss and gain frequency of CNVs across the progeny.
 In general the progeny population shows an accumulation of gains than losses (average gain = 14, average loss = 11). 69% of the progeny have more gains than losses.
