# Supplementary material for: The landscape of inherited and de novo copy number variants in a plasmodium falciparum genetic cross
Source: BMC Genomics. 2011 Sep 22;12:457. doi: 10.1186/1471-2164-12-457 (PMC3191341; doi:10.1186/1471-2164-12-457)
Supplement: Additional file 4 — Hybridization signal distribution in segregating and de novo amplifications. The distribution of the log2ratio of the progeny hybridization signals at segregating and de novo CNV regions were assessed in comparison with that of the parental signal (Dd2/HB3). The positively skewed signal distribution highlights duplicated CNV regions. The clear absence of skewed signal in the Dd2/HB3 parental hybridization compared to that of the positively skewed signal distribution in progeny enabled the identification of de novo amplifications. [file 1471-2164-12-457-S4.PPTX]

## Slide 1
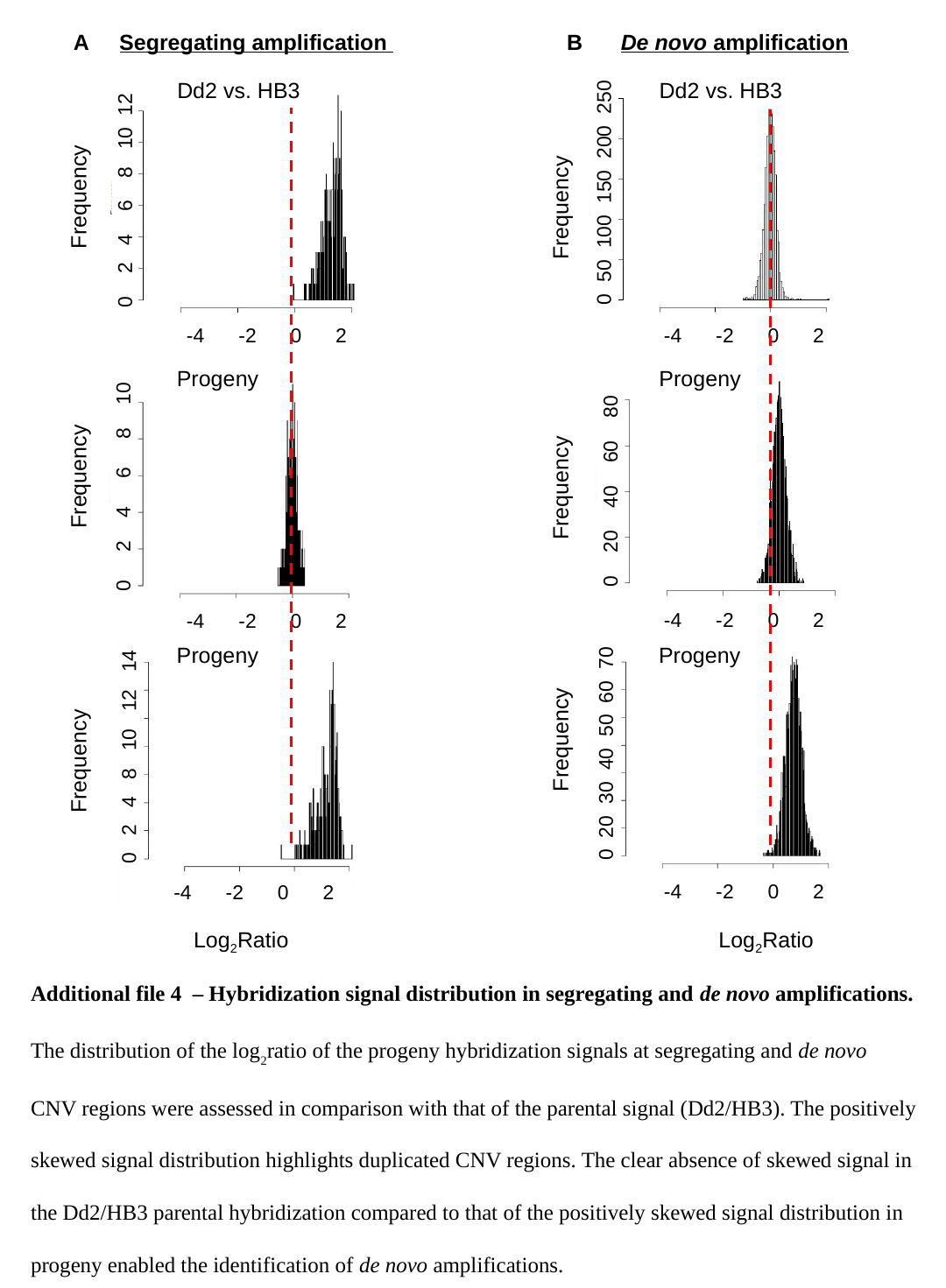

A
Segregating amplification
Dd2 vs. HB3
Frequency
0 2 4 6 8 10 12
-4 -2 0 2
Progeny
Frequency
0 2 4 6 8 10
-4 -2 0 2
Progeny
Frequency
0 2 4 8 10 12 14
-4 -2 0 2
Log2Ratio
B
De novo amplification
Dd2 vs. HB3
0 50 100 150 200 250
Frequency
-4 -2 0 2
Progeny
Frequency
0 20 40 60 80
-4 -2 0 2
Progeny
Frequency
0 20 30 40 50 60 70
-4 -2 0 2
Log2Ratio
Additional file 4 – Hybridization signal distribution in segregating and de novo amplifications.
The distribution of the log2ratio of the progeny hybridization signals at segregating and de novo CNV regions were assessed in comparison with that of the parental signal (Dd2/HB3). The positively skewed signal distribution highlights duplicated CNV regions. The clear absence of skewed signal in the Dd2/HB3 parental hybridization compared to that of the positively skewed signal distribution in progeny enabled the identification of de novo amplifications.
