# Supplementary material for: The landscape of inherited and de novo copy number variants in a plasmodium falciparum genetic cross
Source: BMC Genomics. 2011 Sep 22;12:457. doi: 10.1186/1471-2164-12-457 (PMC3191341; doi:10.1186/1471-2164-12-457)
Supplement: Additional file 5 — Hybridization signal distribution in segregating and de novo deletions. The distribution of the log2ratio of the progeny hybridization signals at segregating and de novo CNV regions were assessed in comparison with the parental signal (Dd2/HB3). The negatively skewed signal distribution highlights deleted CNV regions. The clear absence of skewed signal in the Dd2/HB3 parental hybridization compared to that of the negatively skewed signal distribution in the progeny enabled the identification of de novo deletions. [file 1471-2164-12-457-S5.PPTX]

## Slide 1
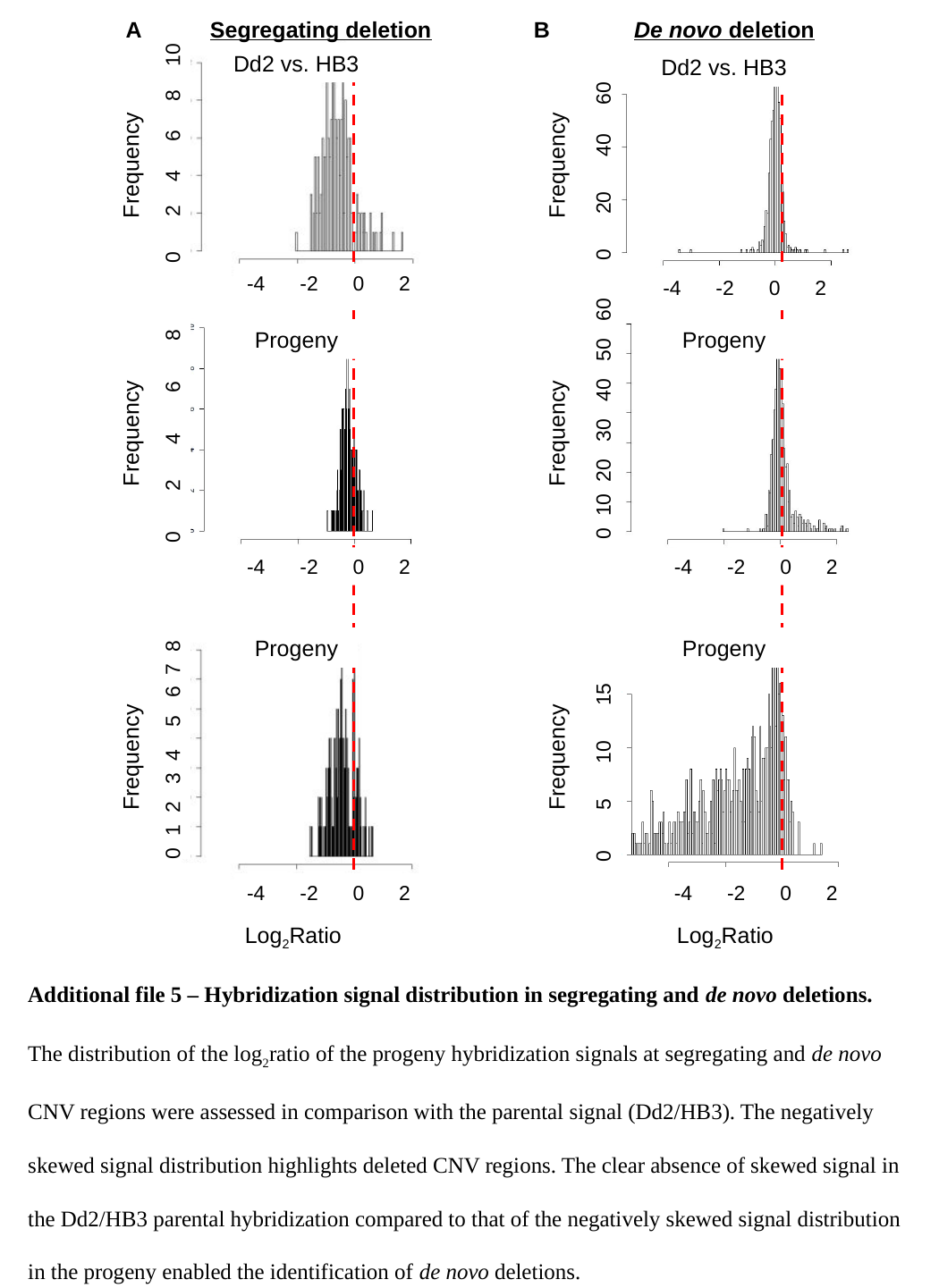

Segregating deletion
A
Dd2 vs. HB3
0 2 4 6 8 10
Frequency
-4 -2 0 2
Progeny
0 2 4 6 8
Frequency
-4 -2 0 2
Progeny
0 1 2 3 4 5 6 7 8
Frequency
-4 -2 0 2
Log2Ratio
B
De novo deletion
Dd2 vs. HB3
Frequency
0 20 40 60
-4 -2 0 2
Progeny
0 10 20 30 40 50 60
Frequency
-4 -2 0 2
Progeny
Frequency
0 5 10 15
-4 -2 0 2
Log2Ratio
Additional file 5 – Hybridization signal distribution in segregating and de novo deletions.
The distribution of the log2ratio of the progeny hybridization signals at segregating and de novo CNV regions were assessed in comparison with the parental signal (Dd2/HB3). The negatively skewed signal distribution highlights deleted CNV regions. The clear absence of skewed signal in the Dd2/HB3 parental hybridization compared to that of the negatively skewed signal distribution in the progeny enabled the identification of de novo deletions.
