# Supplementary material for: The landscape of inherited and de novo copy number variants in a plasmodium falciparum genetic cross
Source: BMC Genomics. 2011 Sep 22;12:457. doi: 10.1186/1471-2164-12-457 (PMC3191341; doi:10.1186/1471-2164-12-457)
Supplement: Additional file 6 — Size distribution of segregating and de novo CNVs. The size distribution of the CNVs was assessed as a percentage of total CNVs in each category. De novo CNVs were predominantly < 10 kb (76%), while segregating CNVs were > 10 kb (55%). In both segregating and de novo CNVs, a small percentage of CNVs were > 100 kb (segregating = 4%, de novo = 2%). [file 1471-2164-12-457-S6.PPTX]

## Slide 1
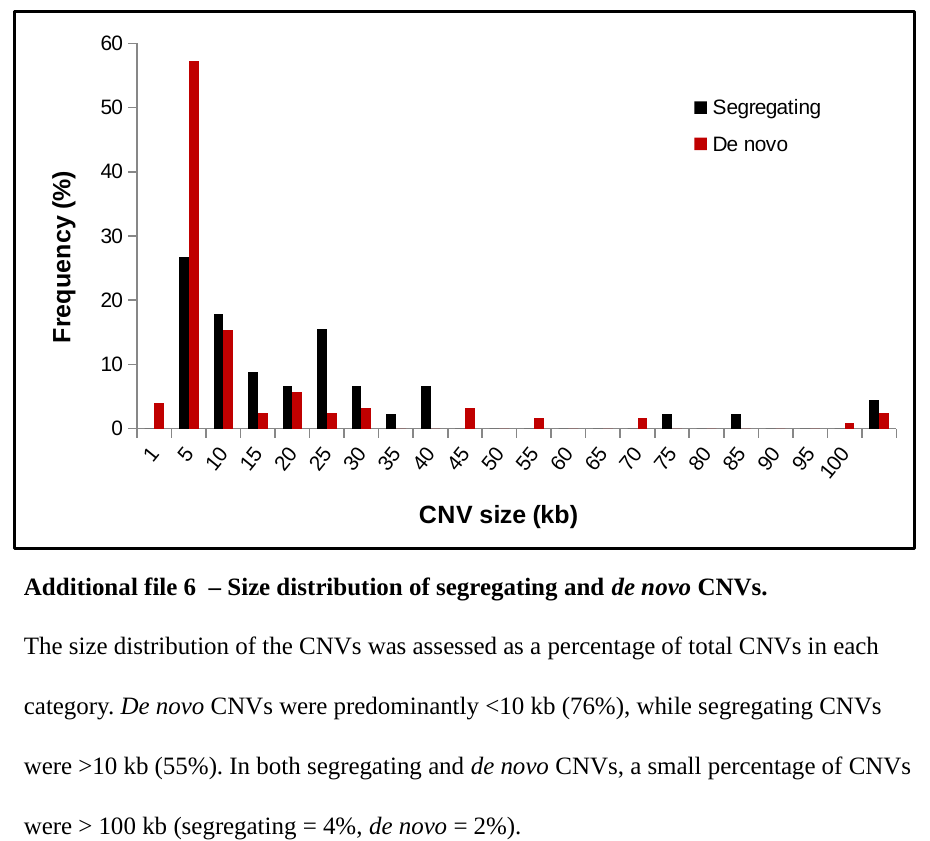

### Chart
| Category | Segregating | De novo |
|---|---|---|
| 1 | 0.0 | 4.0322580645161334 |
| 5 | 26.666666666666668 | 57.25806451612904 |
| 10 | 17.77777777777778 | 15.322580645161334 |
| 15 | 8.88888888888889 | 2.4193548387096775 |
| 20 | 6.666666666666667 | 5.64516129032258 |
| 25 | 15.555555555555587 | 2.4193548387096775 |
| 30 | 6.666666666666667 | 3.2258064516129052 |
| 35 | 2.222222222222223 | 0.0 |
| 40 | 6.666666666666667 | 0.0 |
| 45 | 0.0 | 3.2258064516129052 |
| 50 | 0.0 | 0.0 |
| 55 | 0.0 | 1.6129032258064515 |
| 60 | 0.0 | 0.0 |
| 65 | 0.0 | 0.0 |
| 70 | 0.0 | 1.6129032258064515 |
| 75 | 2.222222222222223 | 0.0 |
| 80 | 0.0 | 0.0 |
| 85 | 2.222222222222223 | 0.0 |
| 90 | 0.0 | 0.0 |
| 95 | 0.0 | 0.0 |
| 100 | 0.0 | 0.8064516129032266 |Additional file 6 – Size distribution of segregating and de novo CNVs.
The size distribution of the CNVs was assessed as a percentage of total CNVs in each category. De novo CNVs were predominantly <10 kb (76%), while segregating CNVs were >10 kb (55%). In both segregating and de novo CNVs, a small percentage of CNVs were > 100 kb (segregating = 4%, de novo = 2%).
