# Supplementary material for: The landscape of inherited and de novo copy number variants in a plasmodium falciparum genetic cross
Source: BMC Genomics. 2011 Sep 22;12:457. doi: 10.1186/1471-2164-12-457 (PMC3191341; doi:10.1186/1471-2164-12-457)
Supplement: Additional file 7 — Gene enrichment within categories of CNVs. [file 1471-2164-12-457-S7.DOCX]

**Additional file 7 - Gene enrichment within categories of CNVs.**

|  | **Term** | **Definition** | | **Corrected**  ***p*-value (FDR) <0.01** |
| --- | --- | --- | --- | --- |
| **Segregating CNVs** | | | | |
| **Molecular** | GO:0003934 | | GTP cyclohydrolase I activity | 0 |
| **Function** | GO:0004240 | mitochondrial processing peptidase activity | | 0 |
|  | GO:0004686 | eukaryotic elongation factor-2 kinase activity | | 0 |
|  | GO:0008757 | SAM-dependent methyltransferase activity | | 0 |
|  | GO:0015559 | multidrug efflux pump activity | | 0 |
|  | GO:0004872 | receptor activity | | 3.2751E-10 |
|  | GO:0005539 | glycosaminoglycan binding | | 8.1566E-08 |
|  | GO:0050839 | cell adhesion molecule binding | | 0.000014735 |
|  | GO:0051082 | unfolded protein binding | | 0.00077561 |
|  | GO:0031072 | heat shock protein binding | | 0.0019936 |
|  | GO:0004012 | phospholipid-translocating ATPase activity | | 0.0037729 |
|  | GO:0004408 | holocytochrome-c synthase activity | | 0.0037729 |
|  | GO:0008143 | poly(A) binding | | 0.0037729 |
|  | GO:0016208 | AMP binding | | 0.0037729 |
|  | GO:0030337 | DNA polymerase processivity factor activity | | 0.0037729 |
| **Biological** | GO:0015917 | aminophospholipid transport | | 0 |
| **Function** | GO:0018063 | cytochrome c-heme linkage | | 0 |
|  | GO:0019438 | aromatic compound biosynthetic process | | 0 |
|  | GO:0009405 | Pathogenesis | | 2.2043E-09 |
|  | GO:0020013 | Resetting | | 0.000019357 |
|  | GO:0016337 | cell-cell adhesion | | 0.000024311 |
|  | GO:0020035 | cytoadherence to microvasculature | | 0.000030777 |
|  | GO:0006631 | fatty acid metabolic process | | 0.0036682 |
|  | GO:0006275 | regulation of DNA replication | | 0.0043429 |
|  | GO:0016458 | gene silencing | | 0.0043429 |
|  | GO:0042493 | response to drug | | 0.0043429 |
|  | GO:0020033 | antigenic variation | | 0.0065862 |
| ***De novo* CNVs** | |  | |  |
| **Molecular** | GO:0003989 | acetyl-CoA carboxylase activity | | 0 |
| **Function** | GO:0004075 | biotin carboxylase activity | | 0 |
|  | GO:0004365 | glyceraldehyde-3-phosphate dehydrogenase activity | | 0 |
|  | GO:0004402 | histone acetyltransferase activity | | 0 |
|  | GO:0004497 | monooxygenase activity | | 0 |
|  | GO:0004776 | succinate-CoA ligase (GDP-forming) activity | | 0 |
|  | GO:0005179 | hormone activity | | 0 |
|  | GO:0008139 | nuclear localization sequence binding | | 0 |
|  | GO:0008382 | iron superoxide dismutase activity | | 0 |
|  | GO:0008426 | protein kinase C inhibitor activity | | 0 |
|  | GO:0008446 | GDP-mannose 4,6-dehydratase activity | | 0 |
|  | GO:0009374 | biotin binding | | 0 |
|  | GO:0015145 | monosaccharide transmembrane transporter activity | | 0 |
|  | GO:0046789 | host cell surface receptor binding | | 0 |
|  | GO:0004872 | receptor activity | | 1.2106E-49 |
|  | GO:0005539 | glycosaminoglycan binding | | 3.1149E-44 |
|  | GO:0050839 | cell adhesion molecule binding | | 2.4923E-40 |
| **Biological** | GO:0006004 | fucose metabolic process | | 0 |
| **Function** | GO:0006086 | acetyl-CoA biosynthetic process from pyruvate | | 0 |
|  | GO:0007131 | meiotic recombination | | 0 |
|  | GO:0007276 | gamete generation | | 0 |
|  | GO:0015749 | monosaccharide transport | | 0 |
|  | GO:0019673 | GDP-mannose metabolic process | | 0 |
|  | GO:0051028 | mRNA transport | | 0 |
|  | GO:0009405 | Pathogenesis | | 1.0912E-34 |
|  | GO:0016337 | cell-cell adhesion | | 5.1119E-34 |
|  | GO:0020013 | Resetting | | 9.3388E-34 |
|  | GO:0020035 | cytoadherence to microvasculature, | | 7.6276E-33 |
|  | GO:0020033 | antigenic variation | | 2.6968E-24 |

All CNVs detected were analyzed for prevalence in molecular function and biological processes using (MADIBA). A hypergeometric *p* value for each annotation was used to evaluate the significance of the annotations [99]. Highly significant categories (*p*<0.01) are given above for both event categories.
