# Supplementary material for: The landscape of inherited and de novo copy number variants in a plasmodium falciparum genetic cross
Source: BMC Genomics. 2011 Sep 22;12:457. doi: 10.1186/1471-2164-12-457 (PMC3191341; doi:10.1186/1471-2164-12-457)
Supplement: Additional file 8 — Hotspots of CNV breakpoints. [file 1471-2164-12-457-S8.DOC]

**Additional file 8 - Hotspots of CNV breakpoints**

| **Chromosome** | **Window** | | **Number** | **Overlapping** |
| --- | --- | --- | --- | --- |
|  | **(kb)** | | **of breakpoints** | **gene IDs** |
| 2 | 30001 | 40000 | 5 | PFB0010w, PFB0015c, PFB0020c, PFB0025c |
| 3 | 30001 | 40000 | 3 | PFC0005w |
| 3 | 120001 | 130000 | 3 | PFC0110w, PFC0115c |
| 4 | 30001 | 40000 | 3 | PFD005w |
| 5 | 1330001 | 1340000 | 3 | PFE1635w, PFE1640w |
| 11 | 2000001 | 2010000 | 3 | PF11_0513, PF11_0514, PF11_0515, PF11_0516 |
| 12 | 1690001 | 1700000 | 4 | PFL1947c, PFL1950w |
| 12 | 2240001 | 2250000 | 3 | PFL2665c |
| 13 | 2860001 | 2870000 | 3 | MAL13P1.356 |

## The genome wide distribution of 340 CNV breakpoints for all CNVs detected within the HB3 x Dd2 cross were analysed in 10 kb non-overlapping window analysis. Hotspots were defined given a random Poisson model. >2 breakpoints per window was highly significant (*p* = 0.00001).
