# Supplementary material for: The landscape of inherited and de novo copy number variants in a plasmodium falciparum genetic cross
Source: BMC Genomics. 2011 Sep 22;12:457. doi: 10.1186/1471-2164-12-457 (PMC3191341; doi:10.1186/1471-2164-12-457)
Supplement: Additional file 9 — Genetic linkage in selected CNV regions. The relationship between linkage position and genome location was assessed by QTL mapping, using relative hybridization signal per probe in segregating CNV regions as a phenotype. Each individual probe signal of segregating CNVs mapped to its closest MS marker in the published linkage map for the HB3 × Dd2 genetic cross [65], highlighting the colinearity of the linkage and physical genome at the CNV regions. The pattern remained true for progeny wide inheritance of A) amplified regions (e.g. Chr 5, boxed in red) as well as, B) deleted regions (e.g. Chr 2, boxed in red). [file 1471-2164-12-457-S9.PPTX]

## Slide 1
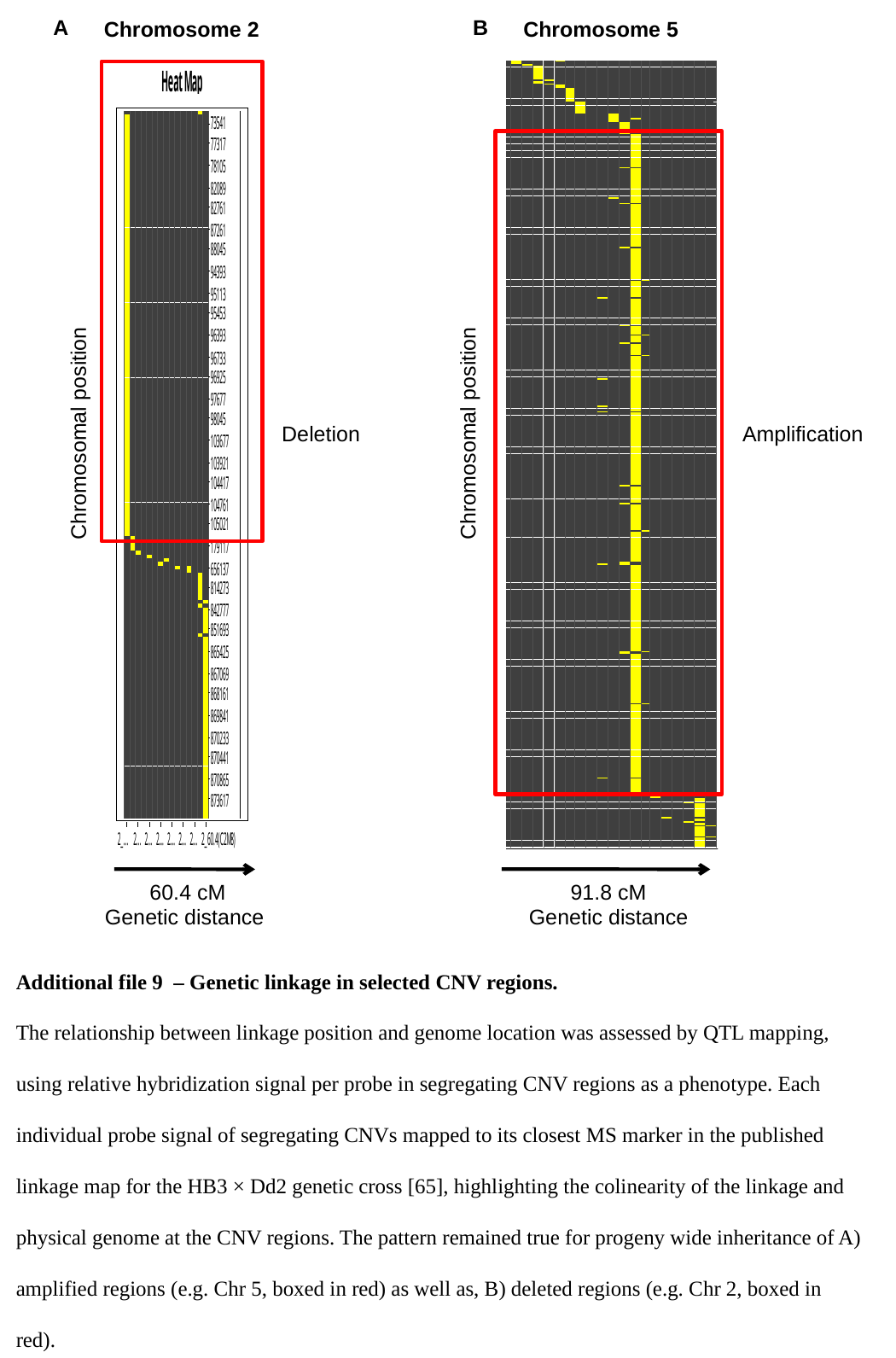

A
Chromosome 2
Chromosomal position
Deletion
 60.4 cM
Genetic distance
B
Chromosome 5
Chromosomal position
Amplification
91.8 cM
Genetic distance
Additional file 9 – Genetic linkage in selected CNV regions.
The relationship between linkage position and genome location was assessed by QTL mapping, using relative hybridization signal per probe in segregating CNV regions as a phenotype. Each individual probe signal of segregating CNVs mapped to its closest MS marker in the published linkage map for the HB3 × Dd2 genetic cross [65], highlighting the colinearity of the linkage and physical genome at the CNV regions. The pattern remained true for progeny wide inheritance of A) amplified regions (e.g. Chr 5, boxed in red) as well as, B) deleted regions (e.g. Chr 2, boxed in red).
