# Supplementary material for: The landscape of inherited and de novo copy number variants in a plasmodium falciparum genetic cross
Source: BMC Genomics. 2011 Sep 22;12:457. doi: 10.1186/1471-2164-12-457 (PMC3191341; doi:10.1186/1471-2164-12-457)
Supplement: Additional file 10 — Allele distribution in segregating CNV regions. We directly examined the parental MS inheritance using the published linkage map for the HB3 × Dd2 genetic cross [65] overlapping the regions of segregating CNVs, in each progeny. (A) The expected number of CNVs was compared to the observed parental allele of the CNV region. We found no evidence for divergence from Mendelian expectation (chi square test, p = 0.99). A few CNVs (e.g. i-v) deviated from this expectation due to lack of marker coverage adjacent to the CNV locus and/or complexity of CNV region in parents or progeny, including two regions that has been previously known to display skewed [53] or complex allele distributions: B) single progeny with a complex CNV overlapping a segregating CNV region (A-ii) and C) complex CNV region in parent genomes (A-iv). Selected CNVs are shown by grey boxes within heat maps (Dd2 parent in column 1) and are highlighted by scatter plots. [file 1471-2164-12-457-S10.PPTX]

## Slide 1
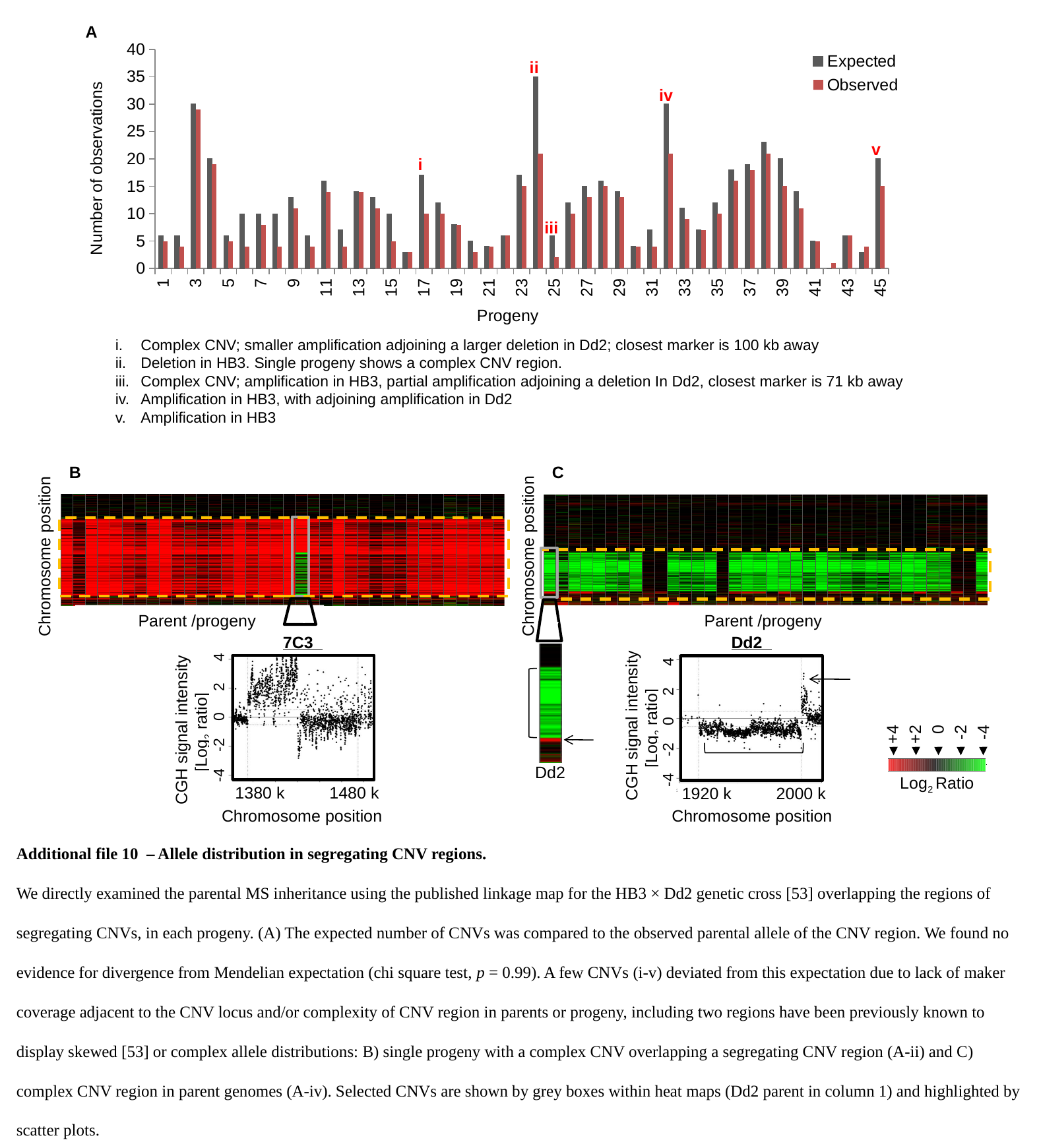

A
### Chart
| Category | Expected | Observed |
|---|---|---|ii
iv
v
i
iii
i. 	Complex CNV; smaller amplification adjoining a larger deletion in Dd2; closest marker is 100 kb away
ii. 	Deletion in HB3. Single progeny shows a complex CNV region.
iii. 	Complex CNV; amplification in HB3, partial amplification adjoining a deletion In Dd2, closest marker is 71 kb away
iv. 	Amplification in HB3, with adjoining amplification in Dd2
v. 	Amplification in HB3
B
Chromosome position
Parent /progeny
C
Chromosome position
Parent /progeny
v
v
7C3
Dd2
Dd2
+4
+2
Log2 Ratio
 0
-2
-4
CGH signal intensity [Log2 ratio]
-4 -2 0 2 4
CGH signal intensity [Log2 ratio]
-4 -2 0 2 4
 1380 k 	1480 k
 1920 k 	2000 k
Chromosome position
Chromosome position
Additional file 10 – Allele distribution in segregating CNV regions.
We directly examined the parental MS inheritance using the published linkage map for the HB3 × Dd2 genetic cross [53] overlapping the regions of segregating CNVs, in each progeny. (A) The expected number of CNVs was compared to the observed parental allele of the CNV region. We found no evidence for divergence from Mendelian expectation (chi square test, p = 0.99). A few CNVs (i-v) deviated from this expectation due to lack of maker coverage adjacent to the CNV locus and/or complexity of CNV region in parents or progeny, including two regions have been previously known to display skewed [53] or complex allele distributions: B) single progeny with a complex CNV overlapping a segregating CNV region (A-ii) and C) complex CNV region in parent genomes (A-iv). Selected CNVs are shown by grey boxes within heat maps (Dd2 parent in column 1) and highlighted by scatter plots.
