# Supplementary material for: The landscape of inherited and de novo copy number variants in a plasmodium falciparum genetic cross
Source: BMC Genomics. 2011 Sep 22;12:457. doi: 10.1186/1471-2164-12-457 (PMC3191341; doi:10.1186/1471-2164-12-457)
Supplement: Additional file 11 — Allele distribution in recurrent de novo CNVs. We directly examined the parental MS inheritance [53] adjacent/overlapping the recurrent de novo CNVs in progeny. (A) Curiously, most CNVs were observed to carry one parental allele in progeny with the CNV. CNVs which were widely recurrent (> 5 progeny) were investigated closely and were discovered to be: (B) segregating regions (boxed in red) within which one of more progeny exhibited overlapping de novo CNV (boxed in gray) and/or (C) segregating complex regions (one or more CNVs in one or both parents). Selected CNVs are shown in boxed regions in the heat maps (Dd2 parent in column 1) and highlighted by the scatter plots. [file 1471-2164-12-457-S11.PPTX]

## Slide 1
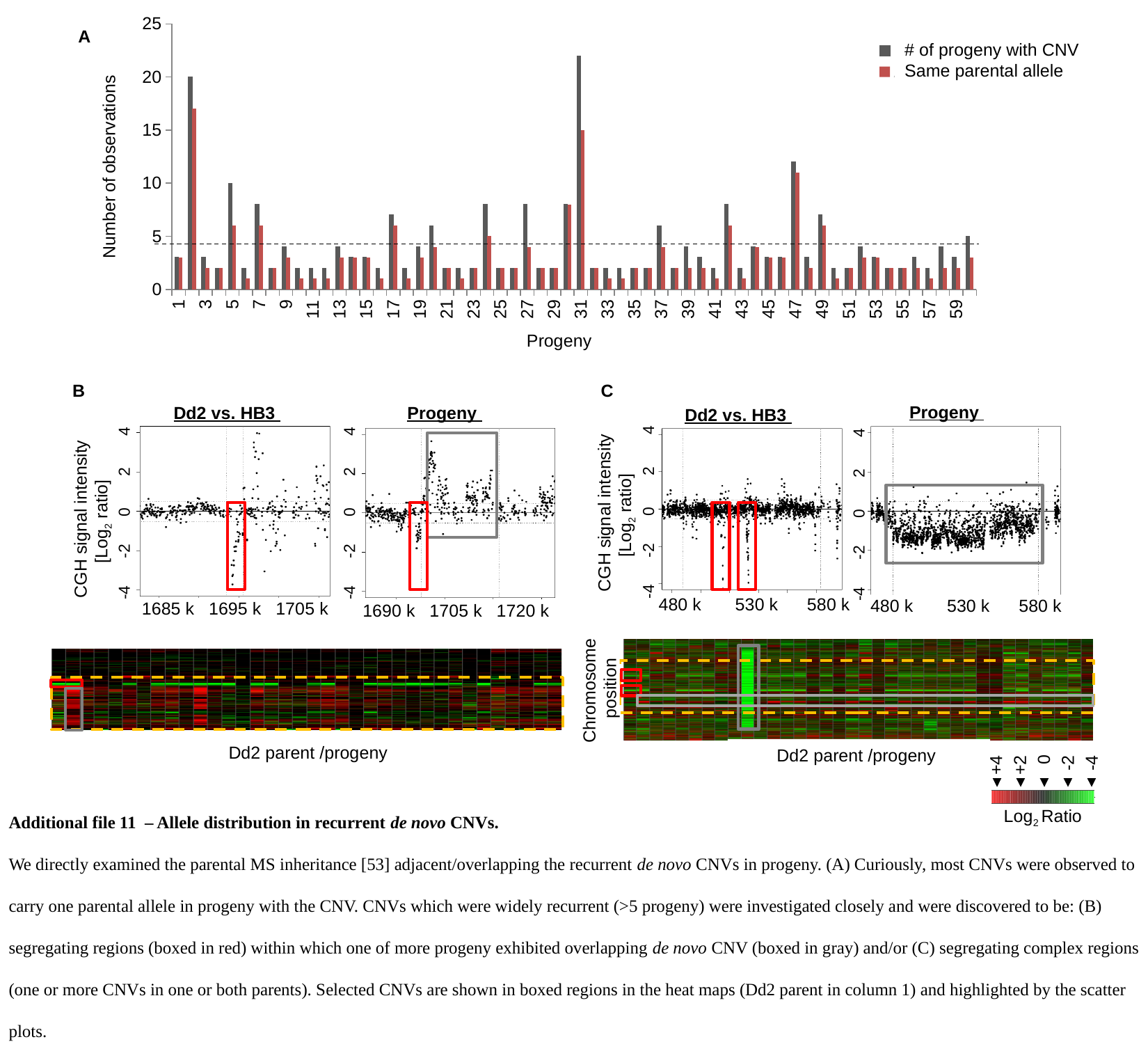

### Chart
| Category | Expected | Allele |
|---|---|---|A
# of progeny with CNV
Same parental allele
B
Dd2 vs. HB3
Progeny
CGH signal intensity [Log2 ratio]
-4 -2 0 2 4
-4 -2 0 2 4
 1685 k 1695 k 1705 k
 1690 k 1705 k 1720 k
Dd2 parent /progeny
C
Progeny
Dd2 vs. HB3
CGH signal intensity [Log2 ratio]
-4 -2 0 2 4
 480 k 530 k 580 k
-4 -2 0 2 4
 480 k 530 k 580 k
Chromosome
 position
Dd2 parent /progeny
+4
+2
Log2 Ratio
 0
-2
-4
Additional file 11 – Allele distribution in recurrent de novo CNVs.
We directly examined the parental MS inheritance [53] adjacent/overlapping the recurrent de novo CNVs in progeny. (A) Curiously, most CNVs were observed to carry one parental allele in progeny with the CNV. CNVs which were widely recurrent (>5 progeny) were investigated closely and were discovered to be: (B) segregating regions (boxed in red) within which one of more progeny exhibited overlapping de novo CNV (boxed in gray) and/or (C) segregating complex regions (one or more CNVs in one or both parents). Selected CNVs are shown in boxed regions in the heat maps (Dd2 parent in column 1) and highlighted by the scatter plots.
