# Supplementary material for: The landscape of inherited and de novo copy number variants in a plasmodium falciparum genetic cross
Source: BMC Genomics. 2011 Sep 22;12:457. doi: 10.1186/1471-2164-12-457 (PMC3191341; doi:10.1186/1471-2164-12-457)
Supplement: Additional file 12 — Recurrent de novo CNV in a multiallelic region. We directly examined the parental SNP allele inheritance [69] within a recurrent de novo CNV in Chr 12 in the progeny clone 7C126. The de novo CNV region is demarcated by an arrow (A) scatter plot of parent CNV profile, Dd2 parent is compared with HB3 parent; (B) scatter plot of progeny CNV profile, progeny is compared with HB3 parent. (C) SNP map of Chr 12 [69]. Each bar of the SNP map denotes a single SNP allele demarcated by the parent allele. The parent allele is highlighted by red (Dd2) and green (HB3). The SNP allele profile which overlaps the de novo CNV region confirms a HB3 allelic region interspersed within a larger Dd2 allelic region (highlighted by arrow), suggesting a potential gene conversion or double crossover. [file 1471-2164-12-457-S12.PPT]

## Slide 1
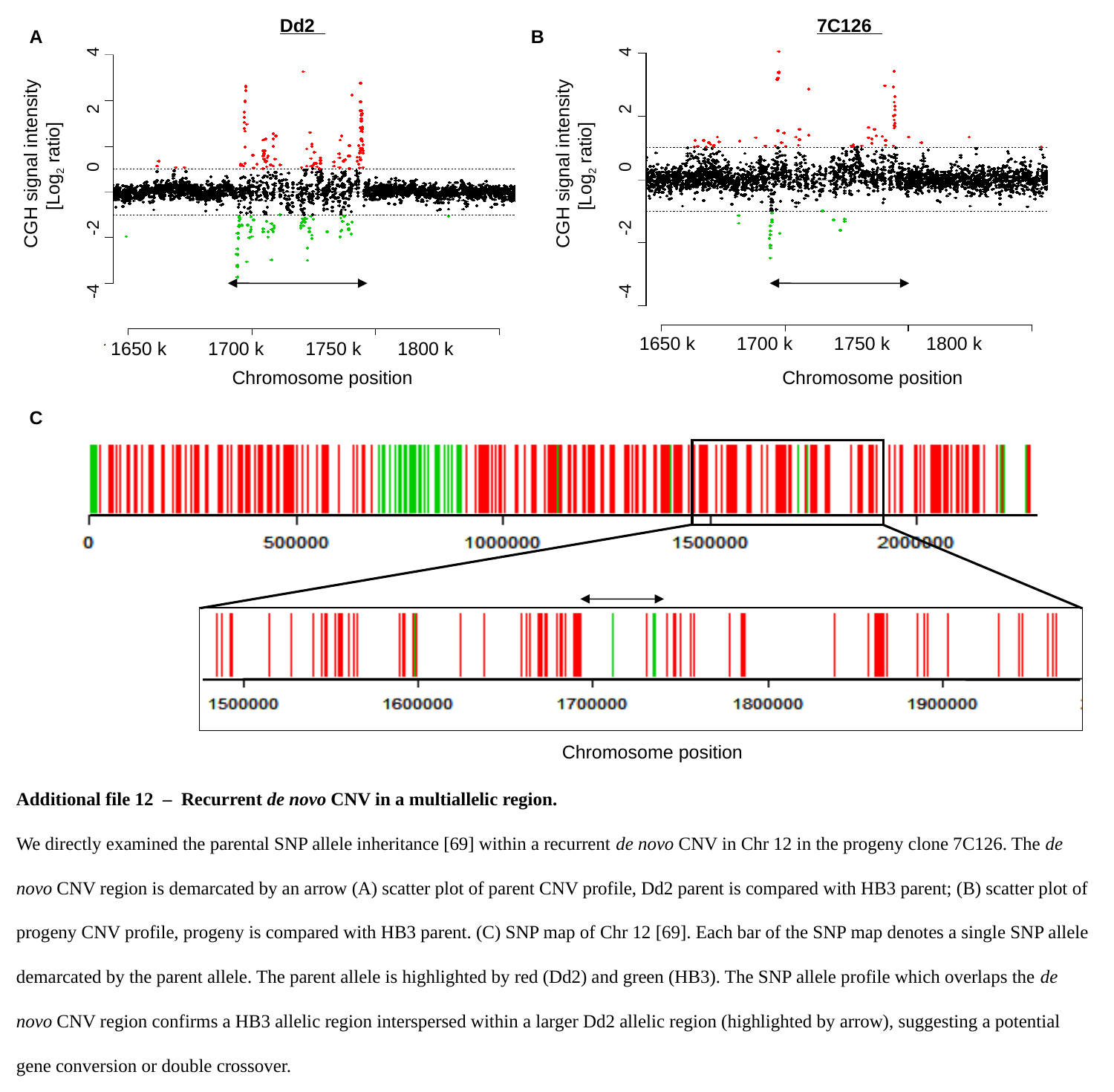

Dd2
7C126
CGH signal intensity [Log2 ratio]
-4 -2 0 2 4
 1650 k 1700 k 1750 k 1800 k
Chromosome position
CGH signal intensity [Log2 ratio]
-4 -2 0 2 4
 1650 k 1700 k 1750 k 1800 k
Chromosome position
Chromosome position
A
B
C
Additional file 12 – Recurrent de novo CNV in a multiallelic region.
We directly examined the parental SNP allele inheritance [69] within a recurrent de novo CNV in Chr 12 in the progeny clone 7C126. The de novo CNV region is demarcated by an arrow (A) scatter plot of parent CNV profile, Dd2 parent is compared with HB3 parent; (B) scatter plot of progeny CNV profile, progeny is compared with HB3 parent. (C) SNP map of Chr 12 [69]. Each bar of the SNP map denotes a single SNP allele demarcated by the parent allele. The parent allele is highlighted by red (Dd2) and green (HB3). The SNP allele profile which overlaps the de novo CNV region confirms a HB3 allelic region interspersed within a larger Dd2 allelic region (highlighted by arrow), suggesting a potential gene conversion or double crossover.
