# Supplementary material for: The landscape of inherited and de novo copy number variants in a plasmodium falciparum genetic cross
Source: BMC Genomics. 2011 Sep 22;12:457. doi: 10.1186/1471-2164-12-457 (PMC3191341; doi:10.1186/1471-2164-12-457)
Supplement: Additional file 14 — Impact of CNVs on gene expression. A previously generated data set of gene expression at 18 hrs in the HB3 × Dd2 progeny population [74] was assessed for impact of CNVs on gene expression. All categories of CNVs resulted in an impact on the gene expression when compared with the gene expression of progeny that do not show CNV in the respective regions. [file 1471-2164-12-457-S14.PPT]

## Slide 1
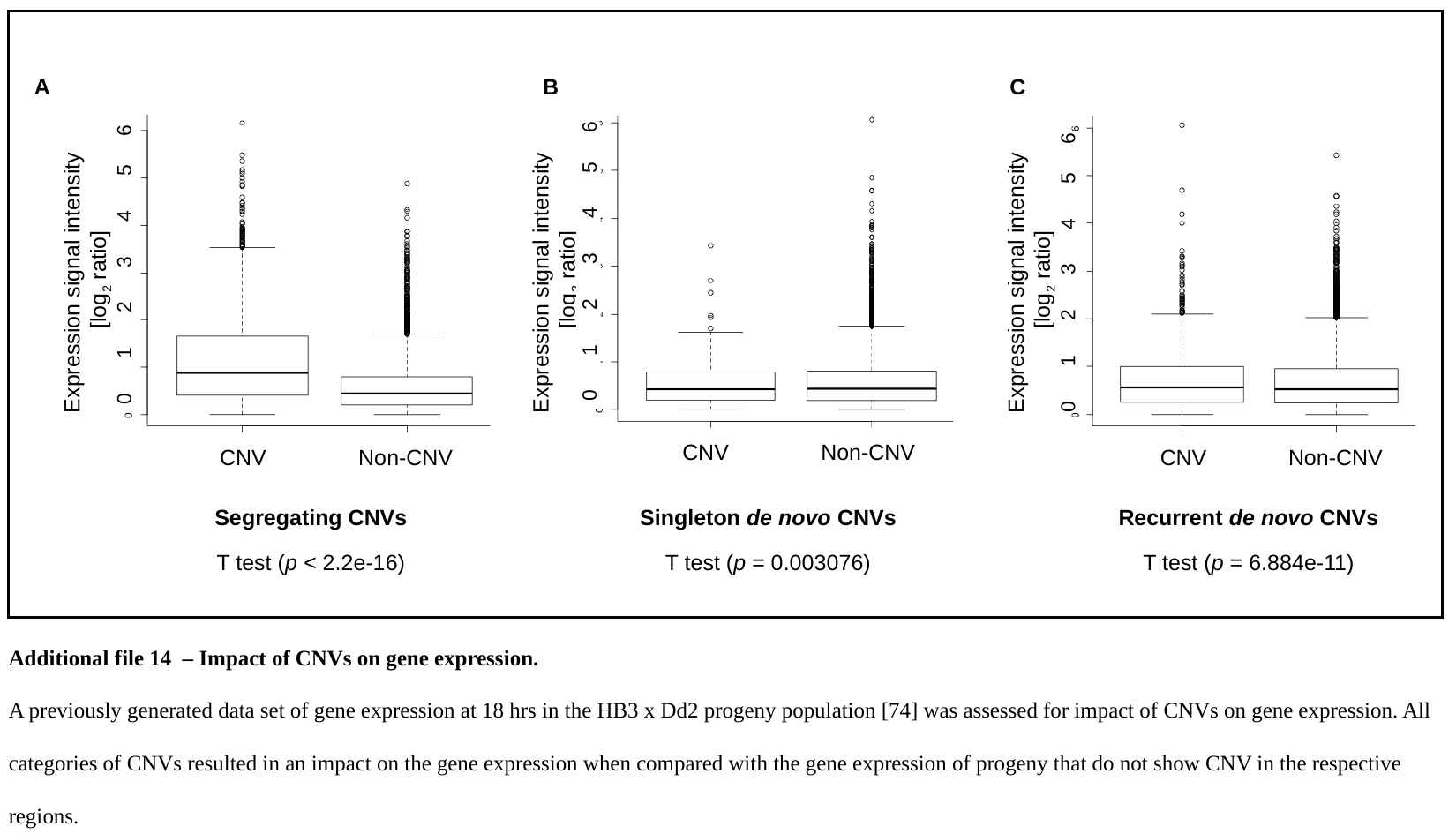

A
B
0 1 2 3 4 5 6
CNV
Non-CNV
Singleton de novo CNVs
T test (p = 0.003076)
C
0 1 2 3 4 5 6
CNV
Non-CNV
Recurrent de novo CNVs
T test (p = 6.884e-11)
0 1 2 3 4 5 6
CNV
Non-CNV
Segregating CNVs
T test (p < 2.2e-16)
Expression signal intensity
[log2 ratio]
Expression signal intensity
[log2 ratio]
Expression signal intensity
[log2 ratio]
Additional file 14 – Impact of CNVs on gene expression.
A previously generated data set of gene expression at 18 hrs in the HB3 x Dd2 progeny population [74] was assessed for impact of CNVs on gene expression. All categories of CNVs resulted in an impact on the gene expression when compared with the gene expression of progeny that do not show CNV in the respective regions.
